# Lateralization of Autonomic Output in Response to Limb-Specific Threat

**DOI:** 10.1523/ENEURO.0011-22.2022

**Published:** 2022-09-07

**Authors:** James H. Kryklywy, Amy Lu, Kevin H. Roberts, Matt Rowan, Rebecca M. Todd

**Affiliations:** 1 Department of Psychology, University of British Columbia, Vancouver, V6T1Z4, Canada; 2 Peter A. Allard School of Law, University of British Columbia, Vancouver, V6T1Z1, Canada; 3 Djavad Mowafaghian Centre for Brain Health, University of British Columbia, Vancouver, V6T1Z3, Canada

**Keywords:** autonomic response, electrodermal activity, fight or flight, lateralization, threat response

## Abstract

In times of stress or danger, the autonomic nervous system (ANS) signals the fight or flight response. A canonical function of ANS activity is to globally mobilize metabolic resources, preparing the organism to respond to threat. Yet a body of research has demonstrated that, rather than displaying a homogenous pattern across the body, autonomic responses to arousing events, as measured through changes in electrodermal activity (EDA), can differ between right and left body locations. Surprisingly, an attempt to identify a function of ANS asymmetry consistent with its metabolic role has not been investigated. In the current study, we investigated whether asymmetric autonomic responses could be induced through limb-specific aversive stimulation. Participants were given mild electric stimulation to either the left or right arm while EDA was monitored bilaterally. In a group-level analyses, an ipsilateral EDA response bias was observed, with increased EDA response in the hand adjacent to the stimulation. This effect was observable in ∼50% of individual participants. These results demonstrate that autonomic output is more complex than canonical interpretations suggest. We suggest that, in stressful situations, autonomic outputs can prepare either the whole-body fight or flight response, or a simply a limb-localized flick, which can effectively neutralize the threat while minimizing global resource consumption. These findings are consistent with recent theories proposing evolutionary leveraging of neural structures organized to mediate sensory responses for processing of cognitive emotional cues.

## Significance Statement

The present study constitutes novel evidence for an autonomic nervous response specific to the side of the body exposed to direct threat. We identify a robust pattern of electrodermal response at the body location that directly receives aversive tactile stimulation. Thus, we demonstrate for the first time in contemporary research that the autonomic nervous system (ANS) is capable of location-specific outputs within single effector organs in response to small scale threat. This extends the canonical view of the role of ANS responses in stressful or dangerous stresses, that of provoking a “fight or flight” response, suggesting a further role of this system: preparation of targeted limb-specific action, i.e., a flick.

## Introduction

An organism’s ability to respond efficiently to threatening situations can mean the difference between survival and death. In the presence of an acute stressor, the autonomic nervous system (ANS), specifically, the sympathetic nervous system, signals the body to prepare for action. Such ANS activation results in increases in cardiac and respiratory outputs, dilation of vasculature in skeletal muscles (in mammals), release of glucocorticoids into the bloodstream, and increased electrodermal activity (EDA; a measure of changes in electrical resistance across the skin because of modulation of the sweat glands; Critchley, 2002; Boucsein, 2012). This “fight or flight” response is highly conserved across vertebrate (Romero and Wingfield, 2016; Romero and Gormally, 2019) and some invertebrate (Shimizu and Okabe, 2007) species and its effective manifestation is critical to the deployment of specific survival behaviors (Blas et al., 2007; Lee and Wang, 2019; Romero and Gormally, 2019; Barrios et al., 2021).

The canonical role of the ANS is that of metabolic mobilization or conservation, demanded by situational cueing (Brener, 1987; Wehrwein et al., 2016; Gibbons, 2019). Emotional arousal in response to relevant events can trigger a sympathetic nervous response (for extended review, see Kreibig, 2010) including increases in sweat gland activity, as approximated through EDA (Vetrugno et al., 2003). Early theories proposed that such responses are homogenous across the entire body (Cannon, 1939). Although the field has moved on, current working assumptions still maintain generalized output to, and responses from, individual effector organs (Jänig and McLachlan, 1992; Folkow, 2000) mediated by a centralized autonomic network (Benarroch, 1993; Damasio, 1998; Valenza et al., 2019). Notably, with respect to the skin (the effector organ monitored during EDA recording), the contemporary division of ANS function still preserves the idea that the skin receives homogenous sympathetic output signaling the need for motor preparedness (Fredrikson et al., 1998; Blakemore and Vuilleumier, 2017; Le et al., 2019). Yet there is a substantial body of research (Richter, 1927; Freixa i Baqué et al., 1984; Picard et al., 2016) demonstrating that asymmetric ANS responses, as measured by changes in EDA, can differ between right and left body locations, albeit not always in a consistent manner (Bjorhei et al., 2019). One potential function of such asymmetry could be a limb-specific response to threat directed to one side of the body, challenging the assumption of global homogeneity. Surprisingly, the question of whether asymmetric ANS responses result from limb-specific threat has been almost wholly neglected.

Historical data from as early as the 1920s (Syz, 1926; Richter, 1927) provides evidence that ANS outputs, specifically, those monitored through EDA, are not always consistent across the body. Examination and explanation of these effects throughout the first half of the 20th century were minimal, relying predominantly on pathologic (Fisher, 1958; Fisher and Cleveland, 1959; Galbrecht et al., 1965) or structural (i.e., lesion dependent; Richter, 1927; Holloway and Parsons, 1969) justification for asymmetry, albeit with some theories pairing EDA asymmetry to degrees of general arousal (Obrist, 1963). While interest in the area of lateralized EDA in both neuro-typical and atypical populations intensified in the 1970s (for review, see Freixa i Baqué et al., 1984), this was paired to the rise of theories evoking gross hemispheric specialization (i.e., right brain vs left brain rhetoric Sperry, 1968; Galaburda et al., 1978; Reeves, 1983), and overlooked what minimal evidence existed for a physiological basis for asymmetric ANS architecture (Fuhrer, 1971). As a consequence, much of this work fell into disrepute alongside the repudiation of the overarching frameworks in which they were nested.

Recently, incidental findings from the field of computer science have reinvigorated interest in EDA asymmetry. In data from wearable electrophysiological recording devices (Poh et al., 2010, 2012; Ayzenberg and Picard, 2014; Sano et al., 2014), collected for the purpose of training computer algorithms to sense, recognize, and respond to human emotional information (Picard, 2002; el Kaliouby et al., 2006; Picard, 2009), asymmetric EDA activity has been observed in response to specific types of emotional situation or arousal (Picard et al., 2016). This work has prompted a secondary resurgence of study on the lateralization of ANS outputs (Banks et al., 2012; Picard et al., 2016; Kasos et al., 2018; Bjorhei et al., 2019) primarily focused on understanding how data from wearable devices can be used to index biomarkers for mental health monitoring (Greene et al., 2016; Ghandeharioun et al., 2017; Mohr et al., 2017; Arza et al., 2019) and clinical impairment (addiction, Carreiro et al., 2015a, b; e.g., autism, Baker et al., 2018; dementia, Kourtis et al., 2019). Stemming from this line of research, the multiple sources of arousal theory (Picard et al., 2016) points to evidence that asymmetric EDA activation can result from ipsilateral signals from “limbic” regions, in particular the amygdalae, linked to stress or emotional arousal. It can also arise from contralateral signals from basal ganglia and premotor regions linked to motor preparedness.

In the context of canonical views of ANS functioning, however, ANS activation as a mechanism to mediate metabolic resource allocation, there is no clear reason why centrally-mediated arousal requiring whole body-responses would elicit greater EDA activity lateralized to one side versus another. Yet, the underlying architecture of the ANS is such that left-right signal variability undeniably occurs (Richter, 1927; Obrist, 1963; Freixa i Baqué et al., 1984; Banks et al., 2012; Picard et al., 2016; Bjorhei et al., 2019). Interestingly, however, almost all previous work displaying asymmetric EDA has involved stimuli that elicit generalized states of arousal not requiring body part-specific responding (Obrist, 1963; situational arousal, Freixa i Baqué and de Bonis, 1983; e.g., face perception, Banks et al., 2012; Picard et al., 2016; high vs low impact stressors, Bjorhei et al., 2019). However, it is unclear how asymmetric responding to generalized arousal could serve a functional purpose within the canonical role of the ANS, that of metabolic conservation; and thus, it is unlikely that these stimuli are the primary developmental motivators of the observed lateralized architecture. Rather, centrally mediated arousal processing is likely leveraging neural architecture based on physical responses to direct threat, where lateralization of response matters. Notably, there is almost no reference made to research conducted on direct tangible limb-specific tactile threat. Yet one largely forgotten historical assay provides preliminary support for the hypothesis that responses to limb-specific threat are lateralized (Fuhrer, 1971). Such preliminary evidence supports the hypothesis that a localized proximal threat to an organism may not require whole body action, but rather a targeted response in the threatened limb. Maximal conservation of metabolic resources would occur if sympathetic activation is evoked in the specific limbs required for the motion that will allow a return to safety, while the rest of the body maintains a state of rest.

The aim of the present study was to investigate lateralized changes in EDA in response to limb-specific aversive stimulation. Specifically, we aimed to identify whether increased sympathetic output is directed to a threatened limb, indicating a potential increase in resource mobilization limited to the site requiring subsequent motor response. To assess lateralization biases in EDA responses, we compared EDA activity from each arm during ipsilateral and contralateral stimulation. Consistent with theories of metabolic conservation in the ANS (Wehrwein et al., 2016; Gibbons, 2019), we predicted that EDA responses would be greater during ipsilateral stimulation, and that this bias would be observable when recording from both the left and right arm.

## Materials and Methods

### Participants

A total of 60 healthy participants (41 females, mean age = 20.45, SD = 2.6) were recruited from the University of British Columbia Psychology Human Subject Pool. Five of the participants indicated they are left-handed. This study was approved by the Behavioural Research Ethics Board (BREB) at the University of British Columbia. Five participants were removed because of insufficient data or incomplete tasks (i.e., <2 completed runs for each hand), and five more were removed because of excessive noise in the recording signal because of inadequate electrode contact or excessive participant motion (i.e., preprocessed data resulted in >50% of individual trials discarded because of noise). Thus, all analyses described below were conducted on the remaining 50 participants (33 females, mean age = 20.48, SD = 2.8).

### Stimulus and apparatus

All psychophysiological recording and stimulation were conducted through an AD Instrument Powerlab 8/35 DAQ device (PL3508) and integrated with the experimental protocol through a custom Python-based program created in Psychopy. Tactile stimulation (max repeat rate = 500 Hz; and pulse width = 1 ms; titrated voltage for each participant) was administered via stimulating bar electrodes (AD Instruments, MLADDF30) connected to an AD Instruments-Stimulus Isolator (FE180). Bar electrodes with a conductive gel (Signagel Electrode Gel) were placed bilaterally on the participant forearms ∼15 cm distal to the elbow and fixed in place with medical tape. Pretrial stimulation was performed to ensure minimal activation of motor units in the arm and hand by the bar electrode. All stimulation events were 50 ms (pulse width = 1 ms, pulse height = 5 V, repeats = 50, repeat rate = 1000 Hz) and administered with a rectangular waveform. Strength of stimulation was titrated independently for each arm until participants reported that it was aversive but not painful. Titration involved increasing stimulus amplitude, beginning at 0.5 mA until a maximum of 10 mA until stimulation elicited a consistent self-report rating of aversive but not painful. Increments varied from 1 to 0.1 mA depending on the previous response to stimulation. Following completion of the titration procedure, stimulation was held constant for the remainder of the experiment.

**Figure 1.**
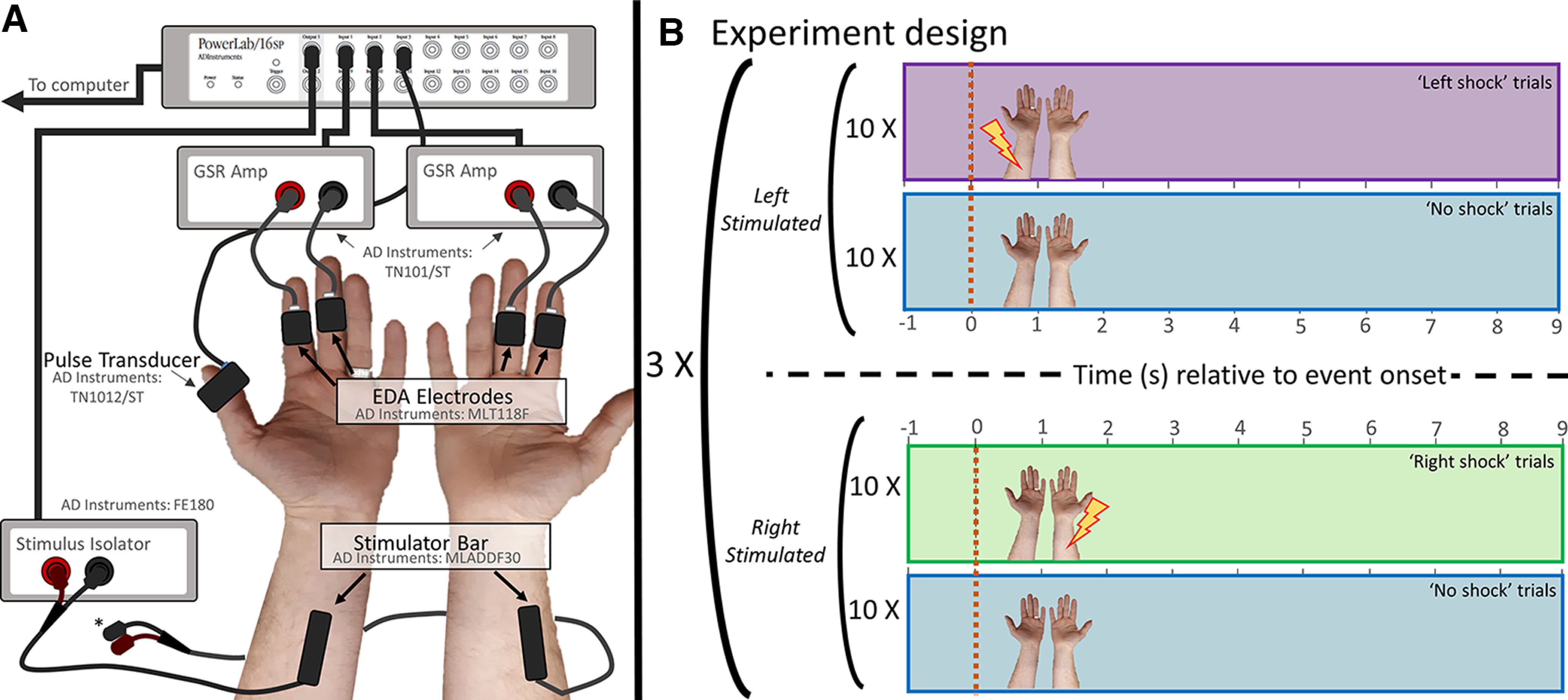
Experimental design. ***A***, Bilateral electrodermal activity (EDA) data were collected from the medial part of the second and third digit on each hand. Stimulator bar electrodes were attached to the proximal half of both the left and right forearm. * Stimulating bar electrodes were only attached to the Stimulus Isolator during experimental blocks in which they were to be used. ***B***, Participants completed six experimental blocks, alternating between blocks targeting stimulation to the left and right forearm (3 blocks per side), respectively, with the starting order counterbalanced across participants. Within each block, participant completed 20 trials: 10 × “Shock” trials, and 10 × “No Shock” trials. “Shock” and “No Shock” trials were presented in random order within blocks and all individual trials had a total duration of 10 seconds. The time line of single trials related to the events of interest (right/left/no shock) is presented at the far right. The dashed line indicates placement of the shock stimulus (i.e., *t* = 0).

EDA was collected simultaneously from two bilateral EDA finger electrodes (AD Instruments, MLT118F), placed over the medial phalange of the middle and index fingers of each hand and amplified by an EDA-amp (AD Instruments, FE116). No conductive gel was used with the EDA electrodes, and they were secured in place with built-in hook-and-loop strapping. In addition, heart rate data were collected with a single finger pulse transducer (AD Instruments, TN1012/ST) connected to the left thumb. Electrode setup generally took less than 5 min before the start of data collection. An attentional visual task was run on PsychoPy v1.90.1, presented on a monitor (BenQ XL Series XL2420B 24 Widescreen; 60 hz) placed ∼60 cm away from the participant. Initial EDA preprocessing was conducted with Labchart 8 (AD Instruments). All data were sampled at 1000 Hz. For a full schematic of the experimental setup, see Figure 1*A*.

### Procedure

Before data collection, participants were asked to sit upright and in a comfortable position, facing away from the experimenter, with their hands placed on a table, to minimize movement throughout the experiment. Participants completed six blocks of a task designed to monitor bilateral EDA response to aversive tactile stimulation, three blocks of shock events targeting each the left and right (e.g., block 1 = [10 shocks left + 10 no shock], randomly ordered; block 2 = [10 shocks right + 10 no shock], randomly ordered). The target of stimulation (left vs right forearm) alternated between blocks, with the initial location counterbalanced across participants. Stimulating electrodes were attached to both arms at the onset of the experiment, with only the electrode corresponding to the desired location of stimulation connected to the Stimulus Isolator for any given experimental block. Over the duration of each block, participants received ten electrical shock events to one arm (the one connected to the isolator), with each event comprised of 50 × 1 ms pulses (see above, Stimulus and apparatus). Trial duration was 10,000 ms, with the shock stimulus administered 1000 ms into this window. “Shock” trials were randomly intermixed with an equivalent number of randomized “No Shock” trials of equal duration. Participants were instructed to remain still for the duration of the experiment and told that no response to the tactile stimuli was required at any point during the task. To minimize participant motion, and maintain engagement, participants were instructed to track the number of color changes in a centrally-presented fixation cross and made a verbal report of this count to the experimenter following each block (range = 40–60 changes per block). Over the duration of the experiment, participants received a maximum of 60 shock events (30 to each arm), as well as 60 “No Shock” events (Fig. 1*B*). Bilateral EDA was collected over the duration of all events. The total duration for all experimental procedures was ∼40 min.

### Preprocessing and analyses

**Figure 2.**
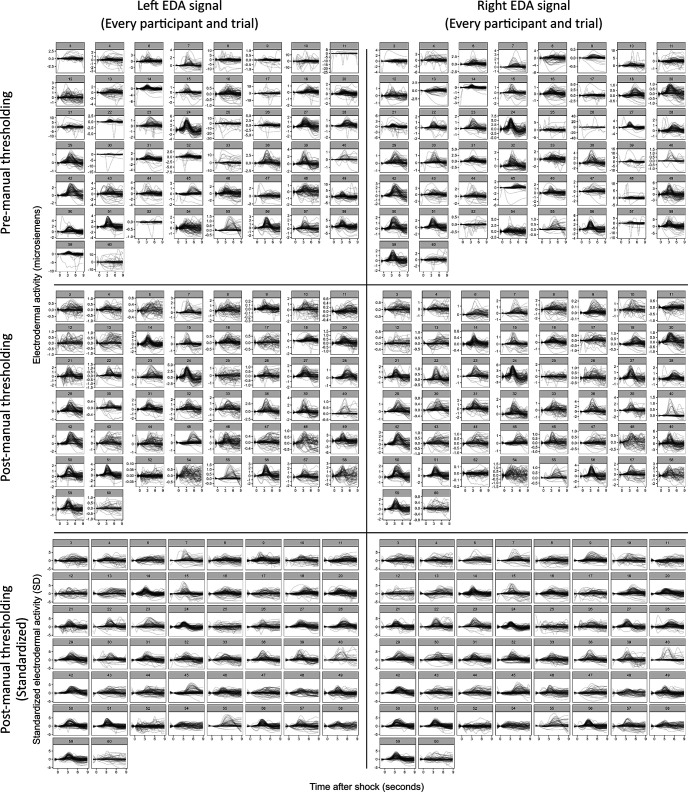
Manual data filtering. Before *z* score standardization, manual data filtering was performed to eliminate trials driven by probable noise in the electrodermal acticity (EDA) signal (e.g., motion artifacts) independently for the right and left recording electrode. Top, Unlabeled EDA signal by trial; baseline corrected, unfiltered. Middle, Unlabeled EDA signal by trial, postmanual filtering. Bottom, Unlabeled EDA signal by trial, post *z* score normalization and an additional baseline correction. SD = standard deviation.

EDA data were exported from Labchart and down-sampled to 100 Hz (from 1000 Hz) to facilitate subsequent analyses. Down-sampling was performed through averaging of 10-ms sample windows (i.e., 10 samples). EDA from the electrode on each hand was subjected to second order Butterworth filter with a bandpass of 0.05–30 Hz. Data were separated into 10-s trial epochs ranging from 1 s before 9 s after each shock/no shock event. To further control for variation in baseline conductance between electrodes and signal drift, all EDA response curves were subject to a baseline correction which standardized EDA at stimulus onset (*t* = 0) for each trial to zero. An additional manual filter of unlabeled trial-by-trial data were conducted to identify an EDA threshold for each electrode channel beyond which the data were most likely attributed to noise (e.g., motion-related artefacts, electrical noise, etc.) in the signal, with these trials to be excluded from further analyses. Manual data filtering eliminated an average of 16.1/117.5 individual trials (range = 0–47). EDA response thresholds were identified independently by two members of the research team (referred to as raters), both blinded to the trial conditions. Interclass correlation estimates found excellent inter-rater reliability (Cronbach’s α = 0.891), and so thresholds identified by Rater 1 were used to filter data before all subsequent analyses (Fig. 2). The data retained after manual thresholding was subject to *z* score standardization for each signal channel (i.e., independently for both the left and right EDA electrodes) within each participant, centered to the signal mean across all trials. A second baseline correction was subsequently performed within each trial to again standardize EDA at stimulus onset (*t* = 0) for each trial to zero (i.e., center the EDA response around signal baseline as opposed to participant mean). This process enabled direct comparison of signal changes across hands and participants, regardless of initial differences in mean amplitude and signal variance. A continuous “Lateralization Bias” index was calculated for each trial by contrasting the standardized right-hand and left-hand EDA (i.e., right hand EDA – left hand EDA) for all sampled time points. The resulting signal indicates the relative strength of EDA between electrode locations, with positive values indicating right-biased asymmetry, and negative values indicating left-biased asymmetry.

For group analyses, data from each participant was collapsed across trial type, resulting in three unique conditions (“Left Shock,” “Right Shock,” and “No Shock”). Three series of one-sample *t* tests were conducted comparing the Lateralization Bias for each condition against a test value of 0 (i.e., no bias) at each time point, with all resultant *p* values subject to a false discovery rate correction (Benjamini and Hochberg, 1995). For single-participant analyses, trial events remained separate, and were used as independent repetitions in subsequent inferential tests. A series of one-sample *t* tests compared lateralization biases during left and right shock events to a test value of 0, replicating the analyses conducted at the group level. In addition, to account for the potential reduction in statistical power from group to within-participant analyses, a series of two-sample *t* tests compared lateralization biases between right and left shock conditions for each participant. As with group analyses, all resultant *p* values were subject to a false discovery rate correction.

## Results

**Figure 3.**
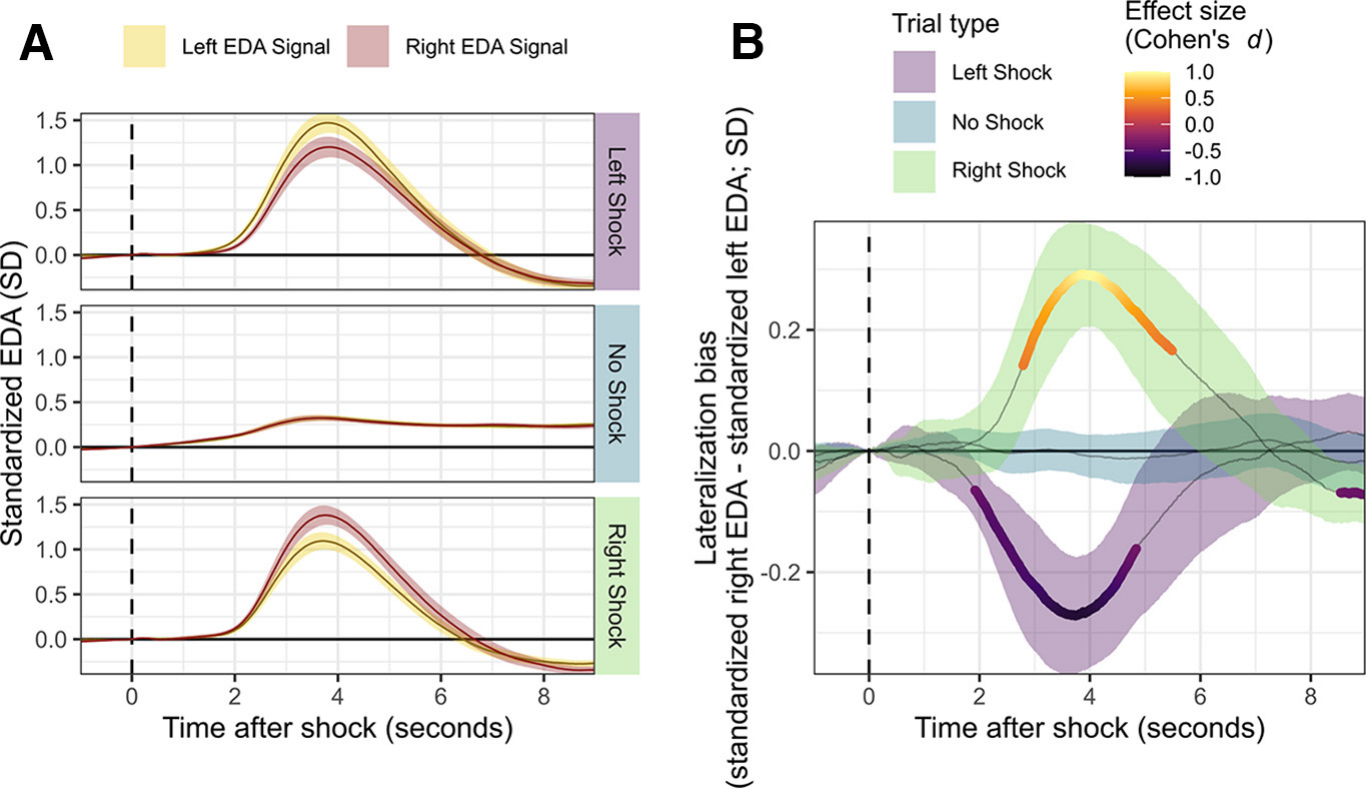
Group-wise contrast of Lateralization Bias in autonomic response. ***A***, Electrodermal activity (EDA) response recorded from both right and left electrodes for each trial condition. For all conditions (“Right Shock,” “Left Shock,” and “No Shock”), the standardized signal is presented for both recording sites (right and left hand) averaged across all trials and participants. Light colored ribbons represent the standard error (SE) across participants at each time point. ***B***, Lateralization biases were defined as [right-hand EDA – left-hand EDA] for each condition (Left Shock, Right Shock, and No Shock). A shift in Lateralization Bias toward either the left (down) or right (up) side indicated a stronger response measured from that location relative to the other side. Significant deviation of these bias scores from a test value of zero (i.e., no bias) are indicated by the highlighted area. Coloring of the highlighted area reflects the effect size (Cohen’s *d*) for contrasts between left-hand and right-hand biases. Light colored ribbons represent the uncorrected 95% confidence interval at each time point.

To assess lateralization biases, we contrasted EDA between the left and right hands in response to mild electric stimulation applied either ipsilaterally or contralaterally to the EDA electrodes (Fig. 3*A*; note that future studies could consider intermixing right and left shock events to minimize potential expectancy biases). To obtain an index of “Lateralization Bias,” *z* score standardized EDA data collected from the left hand was subtracted from that collected from the right hand. These data were then split into three distinct trial types: Left Shock, Right Shock, and No Shock. All reported *p* values have been subject to a false-detection rate correction through the statistical package R (RCoreTeam, 2013), corrected to *p *<* *0.05 (Benjamini and Hochberg, 1995).

### Across-participant analyses

To assess lateralization biases across all participants, a series of one-sample time-locked *t* tests were conducted comparing the observed Lateralization Bias against a test value of zero (i.e., no difference in left vs right side EDA response) for each Left Shock, Right Shock, and No Shock condition averaged within participant. For ipsilateral stimulation, a clear lateralized response bias in both left and right recording sites emerged at ∼2 s after stimulus onset and persisted for ∼3 s (left bias during left shock: significance onset = 1920 ms after trial event, offset = 4840 ms after trial event, |*t*_range(49)_| = 2.69–5.68, all *p *<* *0.05; right bias during right shock: significance onset = 2790 ms after trial event, significance offset = 5490 ms after trial event, |*t*_range(49)_| = 2.68–6.93, all *p *<* *0.05; all times relative to the shock onset): greater EDA was observed at the recording site on the same side as the shock was administered relative to the contralateral recording site. By contrast, Lateralization Bias did not differ from zero at any time point during “No Shock” trials (Fig. 3*B*).

To test whether handedness acted as an independent confound, group-wise analyses were repeated separately for right-handed and left-handed populations in subpopulations. In right-handed participants (*n* = 45), a near identical mirror of the full sample results emerged. Lateralized response bias was observed toward recording sites ipsilateral to tactile stimulation (left bias during left shock: significance onset = 2030 ms, offset = 4650 ms, |*t*_range(49)_| = 2.74–4.88, all *p *<* *0.05; right bias during right shock: significance onset = 2860 ms, significance offset = 5630 ms, |*t*_range(49)_| = 2.74–7.85, all *p *<* *0.05; all times relative to the shock onset). Lateralization Bias did not differ from zero at any time point during “No Shock” trials. In left-handed participants (*n* = 5), Lateralization Bias did not differ from zero at any time point for any condition (all *p *>* *0.05). Because of the limited statistical power of these analyses associated with the reduction in sample size, however, appropriate caution should be taken when interpreting results from left-handed participants.

### Within-participant analyses

**Figure 4.**
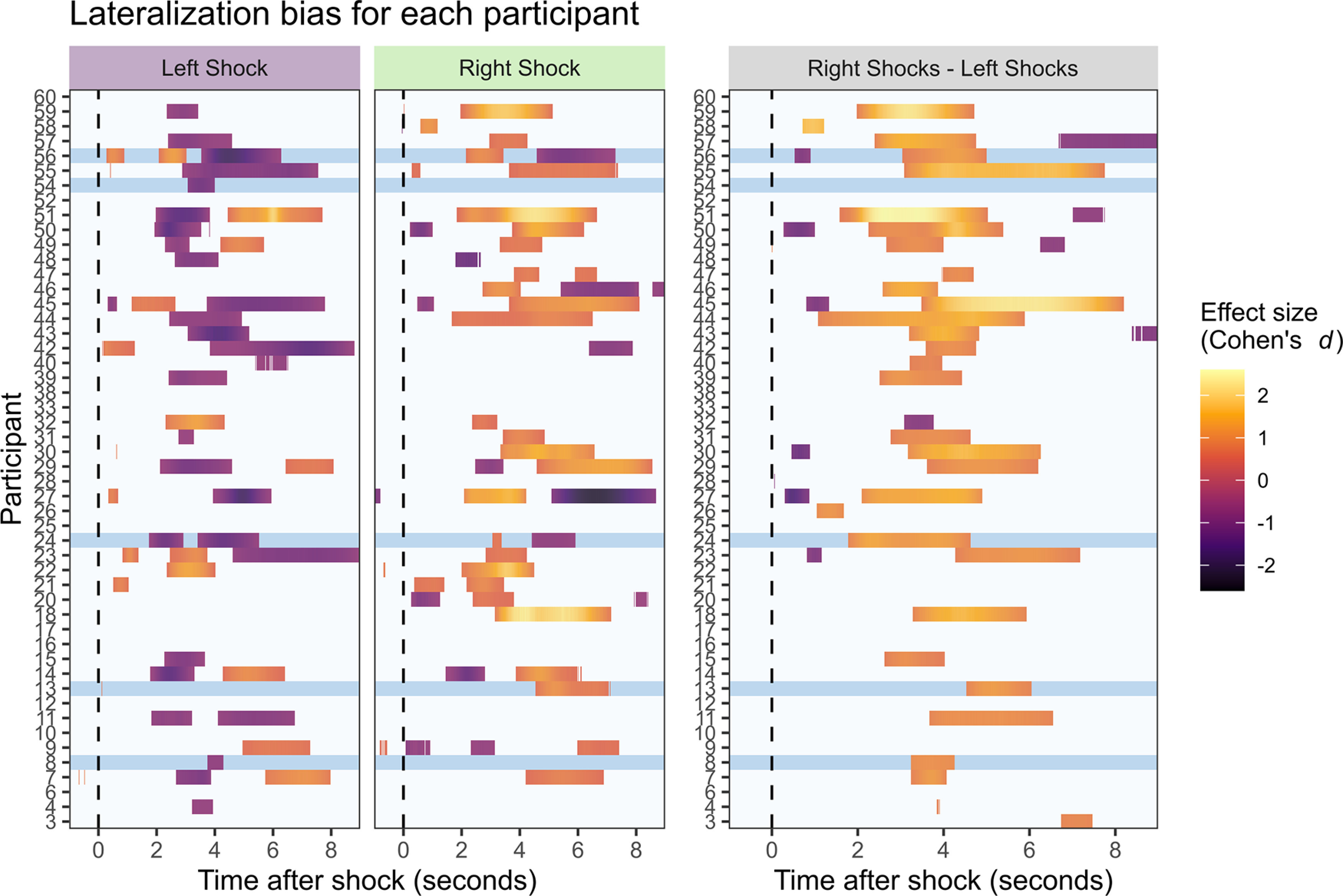
***A***, Lateralization Bias within shock conditions by participant. Lateralization Bias scores for each left and right shock events differing from no bias (i.e., Lateralization Bias = 0) are identified for each participant (row) by trial time course. Cohen’s *d* (calculated with the statistical package R; RCoreTeam, 2013) is presented for all time points with significant lateralization (*p *<* *0.05, FDR corrected), and reflects the effect size for contrasts within each lateralized shock event in relation to a test-bias of zero. ***B***, Lateralization Biases across shock conditions by participant. Differences in Lateralization Bias between left versus right shock events are identified for each participant (row) by trial time course. Cohen’s *d* is indicated for all time points where differences are significant (*p *<* *0.05, FDR corrected) and reflects the effect size for contrasts between left-hand and right-hand biases. Positive values indicate significant right-side bias. Blue banding on all plots indicates left-handed participants.

If lateralized EDA to limb-specific threat is a foundational component of autonomic nervous architecture, this left versus right lateralization should be observable within individual participants as well as across-participant analyses. Accordingly, a similar set of analyses as conducted for group-wise comparisons was conducted at the single participant level. For each participant, a series of one-sided *t* tests compared the Lateralization Bias observed for each trial type to a null value of zero (i.e., no bias in response). Critically, these identified an overall pattern of results mirroring that observed at the group level (Fig. 4*A*). During right-administered shock events, 26 of 50 participants displayed a statistically significant lateralization bias in a direction that was consistent with those observed in the group-wise analyses (i.e., right-side bias). Of the remaining participants, four displayed a left side bias, while 20 displayed no significant bias in either direction. Similarly, during left-administered shock events, an overlapping (but not identical) group of 26 participants displayed a significant left lateralization bias, while of the remaining participants, two displayed a right-side bias and 22 had no significant bias in either direction. Of note, there were a maximum of 30 possible trials for each condition, with an average of 22.5 left shock events and 22.9 right shock events analyzed following data cleaning. This resulted in lower statistical power for the within-participant analyses compared with the group-wise analyses.

To further reinforce the consistency of autonomic lateralization, an additional set of within-participant analyses was performed on trial-by-trial Lateralization Bias for each participant across experimental conditions. In a series of time-locked two-sample *t* tests, significant differences in Lateralization Bias in EDA responses to shock administered to the left versus right forearm were observed in 32 of 50 participants (with 31/32 significant results in the predicted direction; Fig. 4*B*). While the time course of significant differences ranged from as early as 500 ms after stimulus onset to as late as 8000 ms after stimulus onset, the most consistent range was 2000–5000 ms after onset. This is consistent with the range observed in the group analyses, and typical of expected EDA propagation latencies (Boucsein, 2012).

## Discussion

This study investigated whether lateralized EDA would be observed in response to limb-specific tactile stimulation. For both left-administered and right-administered shock, EDA responses were larger at recording sites ipsilateral versus contralateral to the stimulation site. This pattern of results was observed, quite strikingly, in group-wise analyses, while in within-participant analyses, dissociable EDA responses between right and left stimulation were observed in 62% of participants. Together, these findings provide strong evidence that the ANS exhibits robust specificity in EDA, which prioritizes responding in threat adjacent limbs.

While lateralized EDA in response to centrally-mediated, or general, states of arousal (e.g., faces, emotionally salient situations) has been periodically observed throughout the past century (for review of pre-1985 examples, see Freixa i Baqué et al., 1984; also see Poh et al., 2010; Picard et al., 2016; Bjorhei et al., 2019), a potential functional role of this neuro-architectural quirk has remained elusive. Historical explanation of asymmetric autonomic response often evoked now-discredited theories of gross hemispheric lateralization (Sperry, 1968; Galaburda et al., 1978; Reeves, 1983), while recent work has made limited attempts to reconcile EDA lateralization within the canonical ANS function of metabolic conservation (Picard et al., 2016; Bjorhei et al., 2019). The current study demonstrates that lateralized EDA response is not only observed in response to centrally mediated arousal but can also be evoked by limb-specific aversive stimulation. This potentially provides a physiological rationale for the development of asymmetric architecture in the ANS consistent with its canonical role of metabolic resource management. Specifically, increased EDA was observed in both the left and right hands following ipsilateral (vs contralateral) electrical stimulation. This provides evidence for a functional role in threat response for lateralized changes in EDA. We suggest that the sympathetic output of our ANS, the driver of EDA (Vetrugno et al., 2003), prepares the body for a response option beyond the popular alliterative of “fight or flight”; it can ready us to flick.

### Heterogeneity of autonomic output

Evidence of hemisphere-specificity in autonomic responses is consistent with the increasingly prevalent view that ANS output, and particularly ANS output in response to emotional arousal, should not be interpreted as a single measure of balance between global activation/conservation of resources (for review, see Kreibig, 2010). Further, this work demonstrates that heterogeneity of ANS outputs extends beyond differential signaling to separate effector organs, as it also includes differential signaling across body-locations within a single effector organ (i.e., skin). This is consistent with the view that, when a limb-specific motor response is required in response to threat, limb-specific ANS outputs increase local action-preparedness while a state of rest across the rest of the body is preserved. Through this mechanism, the global loss of metabolic resources can be minimized while still ensuring resources are available for adequate behavioral responses to threatening objects or situations (Wehrwein et al., 2016; Gibbons, 2019).

The current work is the first contemporary study to identify threat-localized lateralization in ANS responding and the first to observe it at a single participant level. As early preliminary work in the area (Fuhrer, 1971) was initially overlooked, and later disregarded entirely, much remains to be learned about the specificity of the autonomic response to localized threat. While we provide evidence for local specificity of cutaneous responses to tactile stimulation, it is unclear whether similar spatial heterogeneity also occurs in response to localized threat in other ANS effector organs (e.g., muscle-specific vascular dilation), or in response to localized threats assessed by other modalities (e.g., visual threat). While some autonomic measures may best be regarded as homogenous outputs under heterogeneous control, e.g., it is unlikely that the right lung with be have a greater response the left, other response likely manifest with more concurrent diversity to allow maximal metabolic conservation. For example, similar reasoning on the need to limit motor preparedness would suggest that vascular dilation should also be limited to the threat targeted limb, rather than across the entire body. While the neural architecture for limb-specific vascular responding is well established, localized patterns of dilation are well documented during motor activity and exercise (Wang, 2005; Green, 2009; Green et al., 2017), it is unclear whether this localized vascular responding can be used by the ANS preceding a threat-reduction response as well. It is also unclear whether limb-specific cutaneous activity is observed in response limb-localized nontactile threats, such the sight of a spider approaching the hand. Further investigation into modal specificity required to provoke body-localized changes in EDA would provide further insight into the functionality of such ANS responses, as well as highlight potential central structures involved in mediating these outputs. Additionally, subsequent studies that use threatening object that do not depend on electrical stimulation, a signal also used as the dependent measure in EDA, may be able to tease apart the impact of induced electrical propagation through our peripheral nerves that could confound the current results.

While current results provide strong evidence of limb-localized sympathetic responding in group analyses, this effect was not observed for all individual participants. Indeed, significant unilateral bias toward the threatened limb was observed in just >50% of participants when analyzing data from a single shock location, and just grater than just >60% when analyzing data across shock locations. Furthermore, ∼25% of participants displayed a lateralization bias at some point in the experimental time course that was opposite to that predicted (note: this does not imply a lack of bias in the predicted direction as analyses were conducted independently across time points; see Fig. 4). While we are hesitant to speculate on the route of this of these inconsistencies, as the number of trials and participants limit our ability to perform conclusive individual difference analyses, this is an avenue that would be of interest for future work.

### Development of cognitive arousal systems

A critical contribution of the current work is that we examined lateralized biases in electrodermal responding in response to direct physical threat to a distal sensory target, rather than manipulating or examining circumstances triggering emotional arousal to examine a centrally-mediated cognitive state. The importance of this difference becomes apparent in the context of understanding the biological role and evolutionary development of heterogeneity in cutaneous ANS functioning. To date, contemporary work in the field has largely ignored these questions, focusing instead on either the applied uses of asymmetric electrodermal signaling, including monitoring health and wellness (Carreiro et al., 2015b; Greene et al., 2016; Ghandeharioun et al., 2017; Arza et al., 2019; Kourtis et al., 2019), or the cognitive states during which they manifest (Picard et al., 2016; Mohr et al., 2017). While these are important practical considerations when applying EDA signaling to health care concerns, neither provide any reasonable rationale for how the lateralized autonomic system they are describing may have developed.

By demonstrating greater EDA in body areas adjacent to versus distant from tactile threat, we have provided evidence that supports the proposal that the lateralized neural architecture observed in the cutaneous ANS serves a concrete function in efficient threat protection. Furthermore, we propose that the observation of lateralized EDA in response to centrally mediated arousal (situational arousal, Obrist, 1963; faces, Banks et al., 2012; e.g., high vs low impact stressors, Picard et al., 2016; Bjorhei et al., 2019) indicates that at some point during evolutionary development, central arousal systems likely co-opted pathways once dedicated to processing sensory arousal (Kryklywy et al., 2020). While the idea of building cognitive affect processing mechanisms on top of structures involved in sensory affect processing has been outlined for other specific sensory modalities (e.g., oral vs moral disgust; Chapman et al., 2009) and as a modality general evolutionary practice (Kryklywy et al., 2020), these proposals often rely on the interpretation of a shared central representation for these states, e.g., insular representations of disgust, rather than their common influences on peripheral autonomic outputs. The current work, however, highlights a specific example where the affective characteristic shared between general and tactile processes is peripheral in nature, yet only biologically sensible in its tactile manifestation. Autonomic lateralization observed during states of general arousal (Picard et al., 2016) does not have an obvious function related to metabolic management, yet when observed as a response to limb-localized threat, the metabolic benefits are apparent. Additional work investigating the neural underpinning of the ANS modulation to both sensation-provoked and centrally mediated arousals is still required to determine the extent to which these are overlapping processes within the CNS.

## Synthesis

Reviewing Editor: Christoph Michel, Universite de Geneve Decisions are customarily a result of the Reviewing Editor and the peer reviewers coming together and discussing their recommendations until a consensus is reached. When revisions are invited, a fact-based synthesis statement explaining their decision and outlining what is needed to prepare a revision will be listed below. The following reviewer(s) agreed to reveal their identity: Oliver Pabst. Note: If this manuscript was transferred from JNeurosci and a decision was made to accept the manuscript without peer review, a brief statement to this effect will instead be what is listed below.

Both reviewers consider the work relevant and suitable for publication provided that their comments are adequately answered. Given that both reviewers gave very detailed line-by-line comments, we provide the full reviews that should be answered point-by-point.

Reviewer 1:

The authors study the difference between left hand side and right hand side GSR (EDA) by applying electrical shocks to either one of the sides (no shock events as control measurements were done, as well). I really like the idea and agree with the authors that it fills in some gap in studying asymmetry in left and right hand side EDA, since other paper only studied centralized activation.

The paper has good quality. However, I decided for *revise and re-review* since I need clarification in some aspects and since I would like to see some actual recordings.

Please find my comments below.

Introduction

Lines 49-51: *Yet there is a substantial body of research (e.g., 23, 24-26) demonstrating that asymmetric ANS responses as measured by changes in electrodermal activity (EDA) can differ between right and left body locations.*

- I disagree here. Even though asymmetry was observed in 8 out of 25 subjects in specific situations, the overall conclusion of reference 26 is actually that there are no significant differences between left and right hand side EDA.

-> It is stated in the conclusion: *In the present study, a standardized laboratory setup in which a possible loss of an attractive object created a threat-related high-stake situation, did not lead to consistent observation of bilateral asymmetries in EDA.*

- Furthermore, the study in 26 is designed to test the hypothesis of reference 25 (Picard et. al (2016)) and in the conclusion of 26 it is written *Our laboratory-based study did not replicate the retrospective findings reported by Picard et al. (2016) and the present results do therefore not provide support for the previously proposed multiple arousal theory. The current study’s scope and extent, however, is not suitable for a conclusive judgment on a possible multiple arousal theory.*

Lines 67-69: *Recently, incidental findings from the field of computer science have reinvigorated interest in EDA asymmetry. In data from wearable electrophysiological recording devices (37-40)*

- Since *devices* are written in plural: Is it really several electrophysiological recording devices or rather the same or different versions of the same? I am asking since references 37 to 40 are all from the same group (MIT around Picard).

Methods:

Having always a high degree of reproducibility in mind, here are my comments:

Lines 249-250: *All psychophysiological recording and stimulation were conducted through an AD Instrument Powerlab 8/35 DAQ device (PL3508).*

- You used a data aqusition card to control the FE180. The programme that controls the DAQ, is it written by yourself? In which programming language is it written?

Line 253: *(FE180). Bar electrodes with a conductive gel: *

-What type of conductive gel? Company name?

Lines 256-257. All stimulation events were 50 ms (pulse width = 1 ms, pulse height = 5 V, repeats = 50, repeat rate = 1000 Hz) and administered with a rectangular waveform.

- With repeat rate 1000 Hz you mean that each 1ms there is a new pulse, right? It means you have 50 pulses that have a pulse width of 1 ms that start each 1ms. The overall response will look like shown in the figure below (COMMENT: I prepared a figure, but I cannot upload this figure into the this review system.) The point is if you place 50 rectangular signals of 1 ms beside each with 1ms distance that you basically get one overall rectangular signal that has a width of 50 ms, right?

Why not applying a single rectangular pulse of 50 ms in the first place? What is the meaning of this 50 times 1ms pulses, instead?

- What does this 5 V amplitude mean? You are applying electrical current (Ampere, not Volt) of different amplitudes to the subjects.

- Regarding the current amplitude, it is not so clear: So you increased the current in an initial phase until the test subjects had the aversive but not painful feeling and then you used this constant amplitude in the actual test, right? So in the actual test, the amplitude kept constant, right?

Lines 275-276: *Participants completed six blocks of a task designed to monitor bilaterally EDA response to aversive tactile stimulation.*

- Can you please more specific?

- What are the six blocks of a task?

Lines 276-277. *Over the duration of each block, participants received ten electrical shocks to one arm.*

- Not clear. Is one shock equal to one stimulating event (50 pulses that last 1ms each)?

Line 277: *The target of stimulation (left vs. right forearm) alternated between blocks,*

The used current stimulator (AD Instruments-Stimulus Isolator 253 (FE180)) has only one channel. Did you have two stimulating devices or did you have to reconnect the electrodes when you alternated between left and right arm? You placed two pairs of bar electrodes on the left and the right arm each, right?

Lines 278- 279: *Shock events were spaced 10,000ms apart and intermixed with an equivalent number of *No Shock* trials.*

- Please clarify what exactly you mean by intermixed. Please mention the exact time course of what was happening during the whole experiment. A time line or figure would help understanding a lot.

Lines 282-284: *To minimize participant motion, and maintain engagement, participants were instructed to track the number of colour changes in a centrally presented fixation cross and made a verbal report of this count to the experimenter following each block (range = 40-60 changes per block).*

- It seems like this change in color was not supposed to elicit some EDA responses, right? So, during non-shock events you basically just record baseline EDA without any (elicited) EDA responses, is that correct?

- Please clarify block. Was one block, 10 times a shocking event with no shocking events in between? During that time there happened 40 to 60 color changes, is that correct? So in total, the experiment contained 6 blocks (3 blocks containing left hand shocks, 3 blocks containing right hand shocks), right? Please clarify in the paper. It is not so easy to understand.

Lines 289-290: *EDA data was exported from Labchart and down-sampled to 100 Hz (from 1000 Hz) to facilitate subsequent analyses.*

- How was the downsampling done? Each 10th sample or averaging over 10 samples?

Lines 293-294: *GSR thresholds were identified independently by two unique raters.*

- Not clear. Was it done manually by to different persons (rater= person?) or were two different programmes (rater = software) used? If the former, what are the experiences of the persons with respect to analyzing EDA data.

- How do you define GSR threshold? Is it based on the amplitude? Do you say if the amplitude exceeds amount x, then it is considered a response?

Lines 263-265: *EDA was collected bilaterally from by galvanic skin response (GSR) finger electrodes (AD Instruments: MLT118F) placed on the middle and index fingers of each hand and amplified by a GSR-amp (AD Instruments: FE116)*.

- Please specify: It looks like the GSR-amp (AD Instruments: FE116) has only one recording channel. Did you use two GSR-amps of this type to collect EDA bilaterally? I assume that you mean by bilateral recordings that you measured simultaneously on both sides. Please correct me if not and clarify in the paper.

Results

I would strongly recommend that you add time plots of at least one subject of the recorded GSR, preferably showing left and right hand side for the shock events and the non-shock events to the paper. This will give more trust in the results since the reader can see actual recordings and it helps a lot for the understanding. If you do not want to add these plots in the main paper, putting these into the appendix would be sufficient. I would like to see some of the recordings over time.

Figure 1: I am having a hard time to interpret the meaning of figure 1.The (standardized) difference between left hand and right hand EDA looks basically like and EDA response. Since it is the difference that is shown, I am missing some information here: Generally speaking, does it mean that during shock events one side did not elicit a response and the other side did. Alternatively, did both side elicit EDA responses but one side was stronger than the other? How is it for different subjects? Again if you would make some raw data (EDA time plots) available it would be easier to interpret your results.

Lines 113-114: *z -score standardized GSR data collected from the left hand was subtracted from that collected from the right hand.*

You mentioned the *z-score standardized GSR data*. However, I am still not sure on what the standardization is based on. Is it based on the tonic component of the GSR, i.e. the conductance level during resting? If so, how did you determine the basic conductance level? If not, please specify otherwise.

In general: Did you account for the basic conductance level? Differences in responses may occur just due to different baselines.

Why are standard EDA scores (for example, amplitudes of responses, number of responses, onset time) are not used for comparison? With the EDA scores you could also do a direct comparison.

Figure 2: Cohens d. Please specify how you determined Cohens d. Did you use a software (and if yes, which software did you use?). Preferably, write something more about Cohens d in the methods or the results. At least it would be nice if you would discuss the meaning of the numbers or what the results actual mean.

I am still somewhat unclear on what you based your interpretation on. It might be important to look into actual EDA responses (phasic component). Did you actual look whether your data contained actual EDA responses (maybe that is what you mean in lines 293 to 294, see my comment above) and made your comparison based on real responses? One possible interpretation to which I came: You always use the 10000ms time windows and at the beginning of each window you apply a shock (in shock events) and I am assuming that this shock is assumed to elicit a response. In the end you more less look into actual responses. Is that correct? If yes, please make it clear in the text. In addition, how is it for the non-shocking events? Are there responses?

Just a side comment: I am not sure about the common practice in eNeuro but I am used to journals in which there must be a data availability statement. I think sharing data makes a paper more transparent . However, I am not demanding this here and I leave this decision to the authors/the editor.

Discussion,

The discussion puts the made findings into broader perspective. Which is nice (partly maybe too speculative).

However, not sure how to describe it but I a missing some study related content in the discussion. How does the obtained data support your conclusions? For example, what does Figure 1 really tell us and how is it relevant for the conclusion. Were there some interesting observations that you made in single subjects? Were there subjects that had lateralized differences if you applied the shocks to one side but not if you applied the shocks to other side and so on? How relevant is the time difference? And: as mentioned above. How to interpret the difference between left hand and right hand side that you observed (figure 1)? Is the difference based on no response vs. response or is it based stronger response and weaker response. Actually, one thought that I have now: Since you standardize your measured conductance (GSR) could it be that the conductance level actual alters the results?

Lines 154-158: *EDA responses were larger at recording sites ipsilateral vs. contralateral to the stimulation site. This pattern of results was observed - quite strikingly -in group-wise analyses as well as at an individual subject level in more than half of all participants. Together, these findings provide strong evidence that the autonomic nervous system (ANS) exhibits robust specificity in EDA, which prioritizes responding in threat adjacent limbs.*

- More than half of all participants sounds like around half of all participants (I guess it is 31 out of 50 as you mentioned in lines 146-147).

If I understand correctly, 19 out 50 subjects do not show these difference. I am missing some discussion about it. Can we say that there is *Strong evidence (that) the ANS exhibits robust specificity in EDA* or must this interpretation a little more differentiated? Any idea why this asymmetry occurs for some subjects, while it does not for others? Could it be subject specific? Could there be a component in your study design that is accountable for differences. As mentioned before I am really missing these type of reflections.

Reviewer 2:

The authors propose a novel experimental design to test a hypothesis on the lateralization of EDA. This represents an important contribution to the field. As the authors note, the field of EDA research has been plagued by a dearth of research measuring both sides of the body simultaneously. The use of electrical stimulation (shocks) to generate consistent and reproducible skin conductance responses (SCRs) is an excellent choice. The resultant participant size of n=50 is reasonable and the block-trial design is fine (though see methodological concerns below about jittering and participant reporting). The references are appropriate in number, scope, and relevance, and the authors nicely acknowledge the history of EDA research. The writing is clear.

However, there are several methodological omissions that require attention before this article is ready for publication. This study is not currently reproducible with the information given. Assuming this information was recorded and simply accidentally omitted from the paper, I am recommending a revise and resubmit. However, if the information was not recorded or cannot be rectified, the experiment will need to be repeated. I have highlighted these critical edits with double asterisks (**) in the line-by-line edits below.

The authors should also note that these results show that metabolic conservation is one plausible explanation for the lateralized responses. However it is not sufficient or necessary to explain the lateralization. This work would benefit from a paragraph detailing the limitations and outlining future work to tighten the hypothesis testing.

Overall, I look forward to the revision of this work and its contribution to the field!

The following methodological questions/concerns need to be addressed prior to publication:

See additional Line Edits notes marked with double asterisks (**)

What value was z-score standardized? Was it the SCR peak? Were the peaks normally distributed??

How did you normalize the peak height with respect to individual variation?? What were the min/max values across all participants? Was there a baseline phase of the study? What was the range of baseline EDA values during rest? Did you normalize each participant with respect to his/her resting state? (Arguments could be made for all types of normalization, so please justify your choice.)

How long did the participants wait with the electrodes on before the study began? How long did the study last? What gel was used for the EDA electrodes? (And where were they placed, as I asked below.)

You mention the “false discovery rate correction” several times. However, please tell me what your p-value (or alpha) threshold for significance was before correction (presumably p<0.05?), as well as after correction. Did you show that the data were normally distributed?

Were any of your participants “non responders” (typically about 10% of participants in EDA studies have very low signals for unknown reasons)? Did you exclude any participants for having unusually low baseline (or stimulus-driven) EDA?

How are you accounting for the propagation of the electrical stimuli/shock across the nervous system? That is, how do you know that what you’re measuring is SNS activation - not just electrical signals induced from the shock itself?

Did the person know the shock was coming? Could the participant see the examiner? Were there facial or motor cues that could have indicated an upcoming shock? (See notes below about predictably spaced shocks as well.)

Line Edits:

Line 7 of the abstract: “the metabolic function of such ANS asymmetry has not been investigated,” --> However, metabolic function is not being directly investigated in the current study. Rather, metabolic function is part of the inference of the results of the study. Please clarify or remove this line in the abstract and in other places in the text.

Lines 12-13: “This effect was observable in over 50% of individual subjects” --> This result needs to be tempered. While 26/50 is indeed more than 50%, it is not much more than 50% and well within the margin of error/variance for a human-based study. This line blatantly overstates your results. Remove or edit to be more precise.

Lines 17-19: “These findings provide insight into the evolutionary pathway of neural systems processing general arousal by linking observed asymmetry in the peripheral arousal response to a historical leveraging of neural structures organized to mediate responses to localized threat.” --> How do you link the observed EDA asymmetry to a “historical leveraging of neural structures”? This line is not supported in the text. Remove or edit the text to fully support this idea. The paragraph at the end (lines 219-235) are insufficient for this claim, though lines 220-221 are at least more precise (“we have provided evidence that supports the proposal that the lateralized neural architecture observed in the cutaneous ANS serves a concrete function in efficient threat protection.”)

Line 23: “robust pattern electrodermal activity” --> missing “of"

Lines 24-25: “we demonstrate, for the first time in contemporary research, that the ANS is capable of body-localized outputs...” --> The wording here needs to be more precise. The ANS is capable of all sorts of body-localized outputs (heart, lungs, kidney/bladder - any organ is “body-localized” - and the ANS modulates them).

Lines 28-29: As above, these lines should focus on the ipsilateral limb response, not the “body localized” aspect.

Line 37: “EDA; a measure of sweat gland permeability ...” --> Edit for precision. Skin is permeable (arguably), but sweat glands are ducts. The fluid in these ducts are modulated or innervated by the SNS. EDA is a measure of the change in electrical resistance (or really, 1/resistance = conductance) across the skin due to modulation of the sweat glands.

Line 41: “The canonical role of the ANS is the mobilization or conservation” --> weird tense

Line 43: “...including increases in EDA.” --> imprecise. Consider, “ including modulating sweat gland activity, which can be measured or approximated by EDA.”

Line 45-47: “Although...” --> Yes. Nice.

Line 47: “electrodermal-effector organ” --> strange wording choice. Do you just mean the skin? In addition, “electrodermal” refers to the method of measurement, so it does not seem appropriate here.

Line 50: “asymmetric ANS responses -- as measured by changes in electrodermal activity (EDA)” --> Yes. Good.

Lines 56-66 --> nice overview of EDA asymmetry history

Line 112: Kind of strange to switch to GSR after using EDA this whole time. They are synonyms, though EDA is generally considered more accurate (since there are no galvanic effects involved in the production of the signal) and more modern. Consider just using EDA throughout for consistency, even though I know the company uses GSR. (see also Line 263)

Line 115: “p statistics” should probably be “correlation testing” or something similar

Line 121: No hyphen for “Left Shock"

Line 124: To reduce ambiguity, please note whether the p-value you’re presenting is corrected any time you mention it. (e.g., “all p<0.05 after FDR correction”)

Line 124: Duration of R shock = 2.7 s; Duration of L shock = 2.92 s. Why the difference? Are these averages? Was the shock manually controlled? See methodological concerns above.

Line 132-135: “Accordingly, a similar set of analysis as presented for group-wise comparisons were conducted at a single subject level. For each subject, a series of one-sided t-test comparing Lateralization Bias for each individual trial type to null value of zero was conducted.” --> Several typos

**Line 136-138: The revelation that “26 of 50 participants displayed a lateralization bias in a direction that was consistent with those observed in the group-wise analyses” was startling and problematic. 26/50 is half with a margin of error. So half of the participants showed higher SCRs on the right for right shocks? That means half showed higher SCRs on the left for right shocks! It’s likely that I’m missing some important information here, so please explain this result clearly and completely. The paper is not publishable as is with this explanation. There is no lateralization bias if it was nearly 50/50 as to which side of the body showed a higher SCR with a lateralized shock.

Line 149: 2000-5000 ms post onset is what you would expect given known EDA propagation latency, which is useful/important to point out

Line 154: “EDA responses were larger” --> I assume you mean the peak of the skin conductance response (SCR) was larger. However, you have not defined how you are quantifying the “EDA responses”. See methodological concerns above. A figure would be really helpful!

**Lines 155-6: “...at an individual subject level in more than half of all participants.” More than half?? Exactly ONE MORE THAN HALF, if I read lines 136-138 correctly. Do not overstate the results here. Your results and discussions need to clarify why almost half of your participants produced a higher SCR on the contralateral hand prior to publication. I’m hoping it’s just something to do with the methods that was not described well.

Line 162-3: “a plausible functional rationale for this neuro-architectural quirk has remained elusive.” Consider editing for clarity.

Line 164-5: “recent work has not addressed the role of asymmetric autonomic control in the context of metabolic conservation” --> This is a fine point to make, but I’d like some more justification of metabolic conservation in your own paper if you’re arguing that this is one of your major contributions.

Line 170: arm or hand?? Be specific on EDA electrode placement! A figure would be helpful.

Line 179-182: “this work demonstrates that heterogeneity of ANS outputs extends beyond differential signaling to separate effector organs, as it also includes differential signaling across body-locations within a single effector organ (i.e., skin).” --> Good

Line 194: It is unclear what you mean by “homogenous metrics” for cardiac and respiratory outputs, as they are far from homogenous across individuals (different resting heart rates/respiratory rates across individuals, vastly different responses to stimuli and effort, etc.) and are certainly controlled in a dynamic (what you call “heterogeneous” here) way to enable maximal metabolic conservation. The heart and lungs (and almost every major organ) are also jointly controlled by the parasympathetic and sympathetic branches of the nervous system, making them far from “homogenous” (whatever that means) from an ANS point of view. The authors might note that sweat glands are innervated only by the SNS, making the skin a unique organ to analyze SNS activation.

Lines 198-201: “While the neural architecture for limb-specific vascular responding is well established - localized patterns of dilation are well documented during motor activity and exercise (56-58) - it is unclear whether this localized vascular responding can be used by the ANS for motor preparation as well.” --> This sentence is confusing. What do you mean by “vascular responding can be used by the ANS”? Doesn’t the ANS prompt vascular dilation? I am not an expert on vascular dilation, so there may be an afferent signaling pathway I am unaware of, but the sentence is still confusing and the point they are trying to make is not clear.

Lines 202-203: “It is also unclear whether limb-specific cutaneous activity is observed in response to perception of threat through senses other than others, such as the sight of a spider approaching the hand.” --> A few typos make this hard to read and the point could be clearer. The idea of replicating your study using evocative limb-specific visual stimuli - such as a spider approaching one hand versus another - is novel and important. Such a study would decouple the propagation of the electrical signal from the shock from the resultant SCR while maintaining the fear-conditioning study design (which is likely to produce a strong, reliable SCR). However, you need to make this point much clearer in the sentence.

Line 223: “e.g.” is in a weird spot. Not sure if its unconventional location is supposed to mean something...

Lines 228-230: “these proposals often rely on the interpretation of shared patterns of activity within the brain, rather than shared peripheral outputs.” The meaning is unclear here.

Lines 230-232: “where the affective characteristic shared between general and tactile processes is peripheral in nature, yet only biologically sensible in its tactile manifestation.” Meaning unclear.

Lines 232-235: “Additional work investigating the neural underpinning of the ANS modulation to both sensation provoked and centrally mediated arousals is still required to determine the extent to which these are overlapping processes within the central nervous system.” --> Yes! Good!

Line 240: “Five of the subjects indicated they are left-handed.” Given that handedness may have dominant effects in EDA lateralization, the effect of participants should be further analyzed (with handedness as a confound or as a separate analysis).

Line 246: “remaining 50 subjects”. The stats on these final subjects need to be indicated (how many resultant male/female, ages, handedness, etc.)

Line 253: ​​"placed on bilaterally on” --> typo

Line 264: “placed on the middle and index fingers of each hand” --> distal or medial finger placement? Wet electrodes or dry? Was isotonic gel used? Were the electrodes pre-gelled? Were they taped on? (The pressure and stability of the electrode can greatly affect the signal.) You should also mention the size and composition (Ag-AgCl) of the electrodes and the fact that they were wired. I can get some of this information from googling the specific model (great job including that!), but it still should be briefly stated in the text. A figure or photo of the electrode placement and general setup would be helpful.

Line 265: “electrocardiogram (ECG)” --> pulse rate monitor is more accurate.

Line 267: “visual task is run” --> was run

Line 272+: A simple figure/graphic of the procedure would be helpful

**Line 278-9: “Shock events were spaced 10,000 ms apart and intermixed with an equivalent number of ’No Shock’ trials.” --> Were the shocks regularly spaced/predictably timed? There was no jittering?? Or every 10 sec they either got a shock or no shock, and they didn’t know which?? Please clarify, as these details are critical for any experiment using a strong orienting stimulus like a shock (or loud noise, etc.).

Lines 291-294: “manual filter of unlabeled trial-by-trial data was conducted to identify a GSR threshold beyond which the data was most likely attributed to noise in the signal. GSR thresholds were identified independently by two unique raters.” --> Need to describe this process better. What was the “threshold” you used? What was the most common/likely source of noise?

**Line 296+: “z-score standardization” --> It is not clear what is being z-scored. Is it the SCR peak? How were the peaks found (e.g., peak fitting function in some program? manually?)? Are the peaks normally distributed (which would be necessary for z scoring)?? Did you normalize the peak height with respect to individual variation?? Or how did you deal with the inherent signal variation across participants (i.e., participants with a range 0-1 microSiemens vs. 0-20 microSiemens)?

Line 296: “by GSR electrode” --> by a pair of GSR/EDA electrodes

**Line 297-300: “For each independent event (i.e., all shock and no shock events), we calculated the right hand GSR - left hand GSR for each time point, resulting in a continuous ’Lateralization Bias’ index for each event” --> This line is unclear. A continuous Lateralization Bias suggests you are using raw values, but then you are referring to some sort of “event”. Is that the stimulus/shock? Or the peak SCR?? Please clarify.

Line 304-5: with all resultant p values subject to a false discovery rate correction.” --> Tell me what the p-value (alpha) was before and after correction.

The following figures would be helpful:

- A figure showing the EDA and stimulus (shock) electrode setup. In particular, please indicate where on the palms/fingers the EDA electrodes were placed, showing the stimulus electrodes in the same photo for scale. A wider photo showing how the participants were seated and/or the general room setup would also be helpful for reproducibility. Include other concurrent measurement equipment, such as the thumb pulse-ox monitor, wires, screens, etc.

- A figure showing examples of raw EDA signals from a few participants. This will help other readers, especially those less familiar with EDA, to get a sense of the scale and variability of an EDA signal. Include at least two representative examples, and noting the variability across individuals, such as “low responders” with signals staying below 2-3 uS versus those whose peaks reach 20 uS.

Thank you for all of your hard work! I look forward to reading the revised paper!

## Author Response

We would like to thank the reviewers for their constructive comments. For transparency and reproducibility we have now included substantially more detail in our descriptions of methods and added two additional figures illustrating 1) the experimental design 2) the time series results for each independent trial, pre and post manual filtering. We have also clarified or qualified a number of points in the Introduction and Discussion as requested by reviewers. Point by point responses are below (indented, italicized).

Reviewer 1:

The authors study the difference between left hand side and right hand side GSR (EDA) by applying electrical shocks to either one of the sides (no shock events as control measurements were done, as well). I really like the idea and agree with the authors that it fills in some gap in studying asymmetry in left and right hand side EDA, since other paper only studied centralized activation.

The paper has good quality. However, I decided for *revise and re-review* since I need clarification in some aspects and since I would like to see some actual recordings.

Please find my comments below.

Introduction

Lines 49-51: *Yet there is a substantial body of research (e.g., 23, 24-26) demonstrating that asymmetric ANS responses as measured by changes in electrodermal activity (EDA) can differ between right and left body locations.*

- I disagree here. Even though asymmetry was observed in 8 out of 25 subjects in specific situations, the overall conclusion of reference 26 is actually that there are no significant differences between left and right hand side EDA.

-> It is stated in the conclusion: *In the present study, a standardized laboratory setup in which a possible loss of an attractive object created a threat-related high-stake situation, did not lead to consistent observation of bilateral asymmetries in EDA.*

- Furthermore, the study in 26 is designed to test the hypothesis of reference 25 (Picard et. al (2016)) and in the conclusion of 26 it is written *Our laboratory-based study did not replicate the retrospective findings reported by Picard et al. (2016) and the present results do therefore not provide support for the previously proposed multiple arousal theory. The current study’s scope and extent, however, is not suitable for a conclusive judgment on a possible multiple arousal theory.*

We have removed the offending reference form this section, instead adding it as a counterpoint to the other work. While we maintain that Ref. 26 (Bjorhei et al., 2019) does display the potential for lateralization (asymmetry observed in 32% of the sample, as noted by the reviewer), we do want to remain respectful to the authors initial interpretation of the work. See lines 55-58.

Lines 67-69: *Recently, incidental findings from the field of computer science have reinvigorated interest in EDA asymmetry. In data from wearable electrophysiological recording devices (37-40)*

- Since *devices* are written in plural: Is it really several electrophysiological recording devices or rather the same or different versions of the same? I am asking since references 37 to 40 are all from the same group (MIT around Picard).

The plural ’devices’ in this context refers to the bilateral recordings performed by these groups - i.e., multiple devices used to record simultaneously - rather than referring to the brand of device used in this research.

Methods:

Having always a high degree of reproducibility in mind, here are my comments:

Lines 249-250: *All psychophysiological recording and stimulation were conducted through an AD Instrument Powerlab 8/35 DAQ device (PL3508).*

- You used a data aqusition card to control the FE180. The programme that controls the DAQ, is it written by yourself? In which programming language is it written?

All experiment coding and control was performed through a custom program written in the python-based platform, Psychopy. This is now stated in lines 136-138.

Line 253: *(FE180). Bar electrodes with a conductive gel: *

-What type of conductive gel? Company name?

The gel was Signagel® Electrode Gel. This is now included in the manuscript at line 141.

Lines 256-257. All stimulation events were 50 ms (pulse width = 1 ms, pulse height = 5 V, repeats = 50, repeat rate = 1000 Hz) and administered with a rectangular waveform.

- With repeat rate 1000 Hz you mean that each 1ms there is a new pulse, right? It means you have 50 pulses that have a pulse width of 1 ms that start each 1ms. The overall response will look like shown in the figure below (COMMENT: I prepared a figure, but I cannot upload this figure into the this review system.) The point is if you place 50 rectangular signals of 1 ms beside each with 1ms distance that you basically get one overall rectangular signal that has a width of 50 ms, right?

Why not applying a single rectangular pulse of 50 ms in the first place? What is the meaning of this 50 times 1ms pulses, instead?

The reviewer is correct that 50 X 1 ms pulses, with a 1000 hz repetition rate does approximate a single 50 ms pulse. This choice of settings allows us to have a 50ms stimulation period that worked within the physical limitations of the hardware. Our stimulus isolator had a maximum pulse duration of only 2.56 ms (see https://m-cdn.adinstruments.com/owners-guides/Stimulators%20-%20Owners%20guide%20-%20Jun%202020.pdf, pg. 45).

- What does this 5 V amplitude mean? You are applying electrical current (Ampere, not Volt) of different amplitudes to the subjects.

For all pulses, voltage was held constant at 5 V. Amperage was titrated by subject to elicit a consistently aversive subjective experience.

- Regarding the current amplitude, it is not so clear: So you increased the current in an initial phase until the test subjects had the aversive but not painful feeling and then you used this constant amplitude in the actual test, right? So in the actual test, the amplitude kept constant, right?

We apologize for the confusion here. The reviewer is correct that following titration, amperage was held constant for the remainder of the experiment. This has been clarified at line 151-152.

Lines 275-276: *Participants completed six blocks of a task designed to monitor bilaterally EDA response to aversive tactile stimulation.*

- Can you please more specific?

In the main experiment, participants completed 6 alternating blocks of left and right shock events. (3 for each side, counterbalancing the order between participants). In each block, participants had 10 separate 10s ’Shock’ events, wherein they received 50ms shock 1s into the trial. These were intermixed with 10 additional ’No shock’ events, also 10s in duration each. Additional details in this section have now been provided (lines 167-177, 182-184), and a new methods figure (Figure 1b) has been created to outline the design. See below for further details.

- What are the six blocks of a task?

The six blocks consisted of three blocks of each left and right targeted shock events (each block containing 10 shock events), alternating between side, and counterbalanced across participants. i.e., Blk. 1 = [10 shocks left + 10 no shock) Blk. 2 = (10 shocks right + 10 no shock) ... Blk. 5 = [10 shocks left + 10 no shock) Blk. 6 = (10 shocks right + 10 no shock)

This has now been revised for clarity. See lines 167-169 and Figure 1b.

Lines 276-277. *Over the duration of each block, participants received ten electrical shocks to one arm.*

- Not clear. Is one shock equal to one stimulating event (50 pulses that last 1ms each)?

Yes, each shock event is 50 x 1 ms pulses. This has now been clarified at line 144-146, 173-175.

Line 277: *The target of stimulation (left vs. right forearm) alternated between blocks,*

The used current stimulator (AD Instruments-Stimulus Isolator 253 (FE180)) has only one channel. Did you have two stimulating devices or did you have to reconnect the electrodes when you alternated between left and right arm? You placed two pairs of bar electrodes on the left and the right arm each, right?

Stimulator bars were attached to each both forearms prior to the onset of the experiment. As noted by the reviewer, with the Stimulus isolator having a single channel, the specific electrode attached to the isolator was alternated between block. This is now described at line 170-173.

Lines 278- 279: *Shock events were spaced 10,000ms apart and intermixed with an equivalent number of *No Shock* trials.*

- Please clarify what exactly you mean by intermixed. Please mention the exact time course of what was happening during the whole experiment. A time line or figure would help understanding a lot.

Within each block, participants were administered 20 trials, each lasting 10s. Of these, 10 trials were paired with shock, while 10 were not. The order of these were randomized for each block. A methods figure is now provided to supplement the written description. See Figure 1b.

Lines 282-284: *To minimize participant motion, and maintain engagement, participants were instructed to track the number of colour changes in a centrally presented fixation cross and made a verbal report of this count to the experimenter following each block (range = 40-60 changes per block).*

- It seems like this change in color was not supposed to elicit some EDA responses, right? So, during non-shock events you basically just record baseline EDA without any (elicited) EDA responses, is that correct?

That is correct. The colour change was not relevant to the variables of interest, but rather a cue to help the participant remain engaged and avoid movement. ’No-shock’ trials were recordings of ’EDA baseline’ of equivalent duration to ’Shock’ trials (i.e., 10 s) independent of any stimulation. See lines 179-184.

- Please clarify block. Was one block, 10 times a shocking event with no shocking events in between? During that time there happened 40 to 60 color changes, is that correct? So in total, the experiment contained 6 blocks (3 blocks containing left hand shocks, 3 blocks containing right hand shocks), right? Please clarify in the paper. It is not so easy to understand.

Yes, for the most part this is correct. One additional detail is that there were also 10 ’no shock’ trials in each block, randomly intermixed with the shocked trials. These details have now been clarified in the written text (see lines 175-177), and are illustrated in a new figure (Figure 1b).

Lines 289-290: *EDA data was exported from Labchart and down-sampled to 100 Hz (from 1000 Hz) to facilitate subsequent analyses.*

- How was the downsampling done? Each 10th sample or averaging over 10 samples?

Down sampling, as performed by the Export function in Labchart, is conducted through decimation (averaging). This has now been clarified at lines 189-190.

Lines 293-294: *GSR thresholds were identified independently by two unique raters.*

- Not clear. Was it done manually by to different persons (rater= person?) or were two different programmes (rater = software) used? If the former, what are the experiences of the persons with respect to analyzing EDA data.

’Rater’ in this clause does refer to separate people. This has now been clarified at line 198-199. Both raters had prior experience working with psychophysiological traces (EEG, EDA). Additional added transparency in this procedure, including a new figure of pre- and post-filtered trial by trial data, is outlined below.

- How do you define GSR threshold? Is it based on the amplitude? Do you say if the amplitude exceeds amount x, then it is considered a response?

GSR thresholds were defined based on amplitude. However, data was not excluded based on not meeting a minimum threshold for response, but rather excluded if it exceeded the amplitude threshold. This is described at lines 195-198.

For transparency, individual subject data pre- and post- manual cleaning for both the right and left recording electrode are now provided in a new figure (Figure 2).

Lines 263-265: *EDA was collected bilaterally from by galvanic skin response (GSR) finger electrodes (AD Instruments: MLT118F) placed on the middle and index fingers of each hand and amplified by a GSR-amp (AD Instruments: FE116)*.

- Please specify: It looks like the GSR-amp (AD Instruments: FE116) has only one recording channel. Did you use two GSR-amps of this type to collect EDA bilaterally? I assume that you mean by bilateral recordings that you measured simultaneously on both sides. Please correct me if not and clarify in the paper.

This is correct. EDA monitoring was performed with simultaneously from two separate GSR electrodes, one placed on each hand. This is now clarified at Line 153-156.

Results

I would strongly recommend that you add time plots of at least one subject of the recorded GSR, preferably showing left and right hand side for the shock events and the non-shock events to the paper. This will give more trust in the results since the reader can see actual recordings and it helps a lot for the understanding. If you do not want to add these plots in the main paper, putting these into the appendix would be sufficient. I would like to see some of the recordings over time.

As noted above, a new figure containing the GSR response of each trial, in each hand, for all participants, both pre- and post-manual thresholding is now provided (Figure 2).

Figure 1: I am having a hard time to interpret the meaning of figure 1. The (standardized) difference between left hand and right hand EDA looks basically like and EDA response. Since it is the difference that is shown, I am missing some information here: Generally speaking, does it mean that during shock events one side did not elicit a response and the other side did. Alternatively, did both side elicit EDA responses but one side was stronger than the other? How is it for different subjects? Again if you would make some raw data (EDA time plots) available it would be easier to interpret your results.

We agree that the Lateralization Bias to both right and left shock events in Figure 1 resembles an isolated EDA response, but this reflects the relative strength of response between each arm (i.e., “both side[s] elicit[ed] EDA responses but one side was stronger than the other”. This is described in the figure caption for Figure 2, as well as in the Methods lines 205-209 and Results at Lines 224-227.

Lines 113-114: *z -score standardized GSR data collected from the left hand was subtracted from that collected from the right hand.*

You mentioned the *z-score standardized GSR data*. However, I am still not sure on what the standardization is based on. Is it based on the tonic component of the GSR, i.e. the conductance level during resting? If so, how did you determine the basic conductance level? If not, please specify otherwise.

In general: Did you account for the basic conductance level? Differences in responses may occur just due to different baselines.

We apologize for this omission in our standardization procedures. After dividing our EDA data into trial epochs, all response curves were baseline corrected by subtracting the conductance level at the trial onset to control for variation in baseline conductance. Subsequent z-score standardization of the data across all trials within each participant for each hand enabled the comparison of signals across hands and people. These details are now provided on Lines 191-205.

Why are standard EDA scores (for example, amplitudes of responses, number of responses, onset time) are not used for comparison? With the EDA scores you could also do a direct comparison.

Figure 2: Cohens d. Please specify how you determined Cohens d. Did you use a software (and if yes, which software did you use?). Preferably, write something more about Cohens d in the methods or the results. At least it would be nice if you would discuss the meaning of the numbers or what the results actual mean.

Cohen’s D was calculated with the statistical software R. This is used as a visual representation of the effect size in the figures for all time points found to be significant to p < 0.05 (FDR corrected). This information, along with additional calculation details, is now indicated in the figure caption for Figure 4 (formerly figure 2).

I am still somewhat unclear on what you based your interpretation on. It might be important to look into actual EDA responses (phasic component). Did you actual look whether your data contained actual EDA responses (maybe that is what you mean in lines 293 to 294, see my comment above) and made your comparison based on real responses?

EDA responses were systematically observed in both hand to both ipsi-and contra-lateral shock events, with lateralization bias dependent more on strength of response rather than Response vs. no response. To illustrate this, Figure 3 (previously Figure 1) has now been expanded to show the EDA from each shock event separately for each hand in addition to the lateralization bias plot.

One possible interpretation to which I came: You always use the 10000ms time windows and at the beginning of each window you apply a shock (in shock events) and I am assuming that this shock is assumed to elicit a response. In the end you more less look into actual responses. Is that correct? If yes, please make it clear in the text. In addition, how is it for the non-shocking events? Are there responses?

No notable EDA responses were observed during ’no-shock’ events. See the new extended Figure 3 (formerly Figure 1) for details.

Just a side comment: I am not sure about the common practice in eNeuro but I am used to journals in which there must be a data availability statement. I think sharing data makes a paper more transparent . However, I am not demanding this here and I leave this decision to the authors/the editor.

All data for this work will be available through a public repository following publication.

Discussion,

The discussion puts the made findings into broader perspective. Which is nice (partly maybe too speculative).

However, not sure how to describe it but I a missing some study related content in the discussion. How does the obtained data support your conclusions? For example, what does Figure 1 really tell us and how is it relevant for the conclusion.

Figure 1 demonstrates that when shocks are on the left forearm, a stronger EDA response was measured on the left side, and vice versa for the right. The figure caption for Figure 3 (previously Figure 1) has been updated to make this more clear.

Were there some interesting observations that you made in single subjects? Were there subjects that had lateralized differences if you applied the shocks to one side but not if you applied the shocks to other side and so on?

Yes. As stated in the results, lines 263-269, 26 participants displayed lateralization bias for right shock events and “an overlapping (but not identical)” [Line 267] group of 26 participants showed lateralization biases to the left shock events.

How relevant is the time difference? And: as mentioned above. How to interpret the difference between left hand and right hand side that you observed (figure 1)?

We do not believe that the time window of significance can or should be interpreted in the current analyses, as all current analyses were performed at single sample point (i.e., each sampled time point was analysed independently) rather than across any time windows (i.e., we did not investigate latency to peak). Significant effects in lateralization biased in EDA were found broadly for both right and left-lateralized threat, with the bulk of the significant sample overlapping between sides.

Is the difference based on no response vs. response or is it based stronger response and weaker response.

As noted above, difference in observed Lateralization Bias is likely based on response vs. stronger response. See extended Figure 3 (formerly Figure 1).

Actually, one thought that I have now: Since you standardize your measured conductance (GSR) could it be that the conductance level actual alters the results?

We are confident that it this is not the case. This would result in a lateralization bias in the same direction for shock events to either hand. Our results have a clear dissociable bias toward the stimulated side. We do apologies for how our omission of reporting in our baseline correction may have muddied the interpretation regarding conductance levels.

Lines 154-158: *EDA responses were larger at recording sites ipsilateral vs. contralateral to the stimulation site. This pattern of results was observed - quite strikingly -in group-wise analyses as well as at an individual subject level in more than half of all participants. Together, these findings provide strong evidence that the autonomic nervous system (ANS) exhibits robust specificity in EDA, which prioritizes responding in threat adjacent limbs.*

- More than half of all participants sounds like around half of all participants (I guess it is 31 out of 50 as you mentioned in lines 146-147).

Yes, this is correct.

If I understand correctly, 19 out 50 subjects do not show these difference. I am missing some discussion about it. Can we say that there is *Strong evidence (that) the ANS exhibits robust specificity in EDA* or must this interpretation a little more differentiated? Any idea why this asymmetry occurs for some subjects, while it does not for others?

We expect that individual subject’s asymmetry remained below the key statistical threshold because we had lower statistical power in the individual subject analyses than group-wise analyses. (lines 269-272)

Could it be subject specific?

Given the nature f the current design, it is difficult to reliably interpret data from single subjects. As mentioned above, analyses conducted at a single subject level have notably lower statistical power than group-wise analyses. This is a very intriguing question, however, moving forward to be tackled with a design more catered to critically outlining individual subject difference.

Could there be a component in your study design that is accountable for differences. As mentioned before I am really missing these type of reflections.

We have too little data from any single subject to make any global inferences about sources of differences in individual response, other that variability in physiological responses is commonly observed around the mean trends reported in group-wise analyses.

Reviewer 2:

The authors propose a novel experimental design to test a hypothesis on the lateralization of EDA. This represents an important contribution to the field. As the authors note, the field of EDA research has been plagued by a dearth of research measuring both sides of the body simultaneously. The use of electrical stimulation (shocks) to generate consistent and reproducible skin conductance responses (SCRs) is an excellent choice. The resultant participant size of n=50 is reasonable and the block-trial design is fine (though see methodological concerns below about jittering and participant reporting). The references are appropriate in number, scope, and relevance, and the authors nicely acknowledge the history of EDA research. The writing is clear.

However, there are several methodological omissions that require attention before this article is ready for publication. This study is not currently reproducible with the information given. Assuming this information was recorded and simply accidentally omitted from the paper, I am recommending a revise and resubmit. However, if the information was not recorded or cannot be rectified, the experiment will need to be repeated. I have highlighted these critical edits with double asterisks (**) in the line-by-line edits below.

The authors should also note that these results show that metabolic conservation is one plausible explanation for the lateralized responses. However it is not sufficient or necessary to explain the lateralization. This work would benefit from a paragraph detailing the limitations and outlining future work to tighten the hypothesis testing.

Overall, I look forward to the revision of this work and its contribution to the field!

The following methodological questions/concerns need to be addressed prior to publication:

See additional Line Edits notes marked with double asterisks (**)

What value was z-score standardized? Was it the SCR peak? Were the peaks normally distributed??

Raw EDA values for each electrode channel were standardized. This was performed after an initial baseline correction and manual filtering of the data. We realize that our description of these practices was limited in our prior manuscript and have made significant effort to increase the details we now provide. Please see line 202-205. SCR peaks were not explicitly analysed in the current experiment, as analyses were conducted at each sampled time point.

How did you normalize the peak height with respect to individual variation?? What were the min/max values across all participants? Was there a baseline phase of the study? What was the range of baseline EDA values during rest? Did you normalize each participant with respect to his/her resting state? (Arguments could be made for all types of normalization, so please justify your choice.)

We apologize for the procedural omissions in our previous reporting. There was no baseline phase for the study. As noted above, an initial baseline correction was performed for each trial epoch that standardized the EDA signal at t = 0 (i.e., stimulus onset) to zero (i.e., we baseline corrected the EDA signal at the trial epoch level by subtracting the EDA level at stimulus onset from the EDA signal across the entire trial). Peak height was subsequently normalized for each recording site through z-score standardization of the resultant signal. Please see line 191-205.

How long did the participants wait with the electrodes on before the study began?

Participant generally waited less than 5 minutes with the electrodes in place prior to beginning the experiment.

How long did the study last?

The study lasted ∼40 minutes, not including time following the study to remove electrodes and wash electrode gels. This is now reported at line 185.

What gel was used for the EDA electrodes? (And where were they placed, as I asked below.)

Gel was not used for the EDA electrodes, as recommended by the manufacturer. Signagel® Electrode Gel was applied to the bar electrode, however, and this is now stated at line 141.

You mention the “false discovery rate correction” several times. However, please tell me what your p-value (or alpha) threshold for significance was before correction (presumably p<0.05?), as well as after correction. Did you show that the data were normally distributed?

All presented p-values have been corrected using an FDR correction to p < 0.05, using the statistical software R. We now state this in the first paragraph of the results (see lines 227-229). The specific uncorrected p-value corresponding to an FDR corrected value of 0.05 depends on the analysis being corrected, For example, in our current Lateralization Bias analyses (a series of one-sample t-test against a comparison value of 0, see Figure 3B) the correspond uncorrected p value is ∼ 0.01.

Were any of your participants “non responders” (typically about 10% of participants in EDA studies have very low signals for unknown reasons)? Did you exclude any participants for having unusually low baseline (or stimulus-driven) EDA?

While some participants did display unusually low signal (See Figure 2, Participant 52 for example), none were excluded from analyses.

How are you accounting for the propagation of the electrical stimuli/shock across the nervous system? That is, how do you know that what you’re measuring is SNS activation - not just electrical signals induced from the shock itself?

This is a very interesting possibility. One avenue that may speak to this is the time course to the observed effect. Initial appraisal of the latencies of response in ipsi- versus contra-lateral EDA electrodes (relative to the stimulated side), as well as in the time course for the response bias manifestation See Figure 2) would suggest that the measured lateralization biases are not caused by electrical propagation. Specifically, given that the shape and latency of response in each hand does not drastically differ when stimulated ipsi- or contra-laterally to the recording site, it suggests that a common mechanism - one that can vary in amplitude - likely underlies both responses. However, without having a threat stimulus in the current design that does not depend on electrical stimulation, it is difficult to conclusively rule out this possibility. We have added the potential of induced electrical propagation as a discussion point, and will enthusiastic pursue it as we move forward in this line of research. See lines 343-346.

Did the person know the shock was coming? Could the participant see the examiner? Were there facial or motor cues that could have indicated an upcoming shock? (See notes below about predictably spaced shocks as well.)

Participants did not have sightline to the experimenter, but rather faced away from them with only a computer monitor and some electrophysiological equipment visible. This is now noted at line 153. Additionally, due to the intermixing of 50% null trials with 50% stimulated trials for each experimental block, shocks were not predictable spaced, but rather occurred intermittently (albeit still occurring at an intervals divisible by 10 s). More details on this are provided below.

Line Edits:

Line 7 of the abstract: “the metabolic function of such ANS asymmetry has not been investigated,” --> However, metabolic function is not being directly investigated in the current study. Rather, metabolic function is part of the inference of the results of the study. Please clarify or remove this line in the abstract and in other places in the text.

We appreciate this note and have now clarified throughout the text that we are not investigating metabolic functions explicitly, but rather a role of autonomic lateralization consistent with its canonical role in metabolic control. See lines 7-8.

Lines 12-13: “This effect was observable in over 50% of individual subjects” --> This result needs to be tempered. While 26/50 is indeed more than 50%, it is not much more than 50% and well within the margin of error/variance for a human-based study. This line blatantly overstates your results. Remove or edit to be more precise.

Our intention washer was not to overstate our results but reflect both set of within subject analyses. While one-sample contrasts (i.e., left OR right bias vs test bias value of zero) identified significance in 26/51, s two sample contrasts (i.e., left bias vs right bias) found significance in 31/50 subjects. That said, to avoid overstating results, we have edited this line to now read “in ∼50% of individual subjects”. See lines 12-13.

Lines 17-19: “These findings provide insight into the evolutionary pathway of neural systems processing general arousal by linking observed asymmetry in the peripheral arousal response to a historical leveraging of neural structures organized to mediate responses to localized threat.” --> How do you link the observed EDA asymmetry to a “historical leveraging of neural structures”? This line is not supported in the text. Remove or edit the text to fully support this idea.

We understand that we may have overstated our position at this point. We have now reworded this section to more accurately reflect what can (and cannot) be interpreted from the current data. See line 17-19.

The paragraph at the end (lines 219-235) are insufficient for this claim, though lines 220-221 are at least more precise (“we have provided evidence that supports the proposal that the lateralized neural architecture observed in the cutaneous ANS serves a concrete function in efficient threat protection.”)

As noted above, we apologize for overstating our position in the abstract, and have modified our writing to better reflect what can be interpreted form the data. Specific (as the reviewer noted above) that the current work is consistent with recent theories on the evolutionary development of emotion systems, rather than garnering completely novel insight into the area. We hope that these revisions are less likely to unintentionally mislead the reader.

Line 23: “robust pattern electrodermal activity” --> missing “of"

Thank you for the catch! The missing “of” has been added.

Lines 24-25: “we demonstrate, for the first time in contemporary research, that the ANS is capable of body-localized outputs...” --> The wording here needs to be more precise. The ANS is capable of all sorts of body-localized outputs (heart, lungs, kidney/bladder - any organ is “body-localized” - and the ANS modulates them).

We have now amended this line to reflect location-specificity within single effector organs (i.e., the skin) rather than body-specific as targeted to a single organ. See lines 24-26.

Lines 28-29: As above, these lines should focus on the ipsilateral limb response, not the “body localized” aspect.

As with above, this line has been edited to no longer reflect the threat targeted response rather than effector organ heterogeneity. See lines 26-29.

Line 37: “EDA; a measure of sweat gland permeability ...” --> Edit for precision. Skin is permeable (arguably), but sweat glands are ducts. The fluid in these ducts are modulated or innervated by the SNS. EDA is a measure of the change in electrical resistance (or really, 1/resistance = conductance) across the skin due to modulation of the sweat glands.

Thank you for this clarification. We have now updated our description of EDA to be more precise and accurate. See lines 37-38.

Line 41: “The canonical role of the ANS is the mobilization or conservation” --> weird tense

This sentence has been reworked to remove the ’weird tense’ phrasing. See line 43-44.

Line 43: “...including increases in EDA.” --> imprecise. Consider, “ including modulating sweat gland activity, which can be measured or approximated by EDA.”

Thank you for this clarification. The phrase has been modified accordingly. See line 46-47

Line 45-47: “Although...” --> Yes. Nice. Thank you.

Line 47: “electrodermal-effector organ” --> strange wording choice. Do you just mean the skin? In addition, “electrodermal” refers to the method of measurement, so it does not seem appropriate here.

Yes, we did just mean the skin, but were attempting orient the reader to its role as an autonomic effector organ. We have reworked this sentence for easier readability. See lines 51-52.

Line 50: “asymmetric ANS responses -- as measured by changes in electrodermal activity (EDA)” --> Yes. Good. Thank you.

Lines 56-66 --> nice overview of EDA asymmetry history Thank you.

Line 112: Kind of strange to switch to GSR after using EDA this whole time. They are synonyms, though EDA is generally considered more accurate (since there are no galvanic effects involved in the production of the signal) and more modern. Consider just using EDA throughout for consistency, even though I know the company uses GSR. (see also Line 263)

The reviewer is correct in noting that this choice was to reflect the trade name for the electrodes used. In light of this suggestion, we have changes all references to GSR to EDA, unless explicitly referring to a product name.

Line 115: “p statistics” should probably be “correlation testing” or something similar

We apologize for confusing wording. We have rephrased the wording to the more commonly used “p-values.”

Line 121: No hyphen for “Left Shock"

The hyphen has been deleted.

Line 124: To reduce ambiguity, please note whether the p-value you’re presenting is corrected any time you mention it. (e.g., “all p<0.05 after FDR correction”)

All presented p-values have been corrected using an FDR correction to p < 0.05. We now state this in the first paragraph of the results (see lines 227-229)

Line 124: Duration of R shock = 2.7 s; Duration of L shock = 2.92 s. Why the difference? Are these averages? Was the shock manually controlled? See methodological concerns above.

We apologize for the lack of clarity in this section. The shock was controlled by a custom program built with Psychopy (see line 136-138). The durations mentioned above do not refer to the shock itself, but rather the time period within the trial window in which the lateralization bias significantly deviates from 0 during each condition (See red highlighted areas in Figure 3b; formerly Figure 1).

Line 132-135: “Accordingly, a similar set of analysis as presented for group-wise comparisons were conducted at a single subject level. For each subject, a series of one-sided t-test comparing Lateralization Bias for each individual trial type to null value of zero was conducted.” --> Several typos

This section has been proofread for errors.

**Line 136-138: The revelation that “26 of 50 participants displayed a lateralization bias in a direction that was consistent with those observed in the group-wise analyses” was startling and problematic. 26/50 is half with a margin of error. So half of the participants showed higher SCRs on the right for right shocks?

No, this is an unfortunate lack of clarity in our reporting. Many participants did not show any significant bias in either direction. Of those not displaying some significant bias to the expected side, 4 participants showed right-hand bias during left hand shock events, and 2 showed left side bias to right shock events (See figure 4a; formerly figure 2). Furthermore, in a 2 sample t-test comparing bias scores to right and left shock events, of 32 participants who displayed significant bias, 31 had significant bias in the predicted direction (see Figure 3b). This has now been revised for clarity. See lines 263-269.

That means half showed higher SCRs on the left for right shocks! It’s likely that I’m missing some important information here, so please explain this result clearly and completely. The paper is not publishable as is with this explanation. There is no lateralization bias if it was nearly 50/50 as to which side of the body showed a higher SCR with a lateralized shock.

We have now updated the results to indicate that some participants had no lateralization bias, rather than a counter-predicted lateralization bias. This can be seen in lines 263-269, as well as in Figure 4.

Line 149: 2000-5000 ms post onset is what you would expect given known EDA propagation latency, which is useful/important to point out.

This consistency has now been noted. See lines 280-281.

Line 154: “EDA responses were larger” --> I assume you mean the peak of the skin conductance response (SCR) was larger. However, you have not defined how you are quantifying the “EDA responses”. See methodological concerns above. A figure would be really helpful!

As noted above, the presented analyses did not focus solely on the peak response but were rather conducted at each sampled timepoint independently. To visualize this, Figure 3 (formerly figure 1) has been expanded to include non-bias scored EDA traces (Figure 3a).

**Lines 155-6: “...at an individual subject level in more than half of all participants.” More than half?? Exactly ONE MORE THAN HALF, if I read lines 136-138 correctly. Do not overstate the results here. Your results and discussions need to clarify why almost half of your participants produced a higher SCR on the contralateral hand prior to publication. I’m hoping it’s just something to do with the methods that was not described well.

As described above, the results description may have presented a false dichotomy for the results (i.e., participant must display bias to the Left OR Right) and omitted the potential of participants who had no significant bias. This has been clarified now in the results (lines 263-269). In addition, while one-sided t-test against no-bias identified predicted significance in 26/50 subjects, two-sided t-tests found that 31/50 participants displayed significant biases between the two shock conditions, with 18/19 remaining subjects having no bias at all. This is notably ’more than half.’

Line 162-3: “a plausible functional rationale for this neuro-architectural quirk has remained elusive.” Consider editing for clarity.

This sentence has been revised for clarity. See line 294-295.

Line 164-5: “recent work has not addressed the role of asymmetric autonomic control in the context of metabolic conservation” --> This is a fine point to make, but I’d like some more justification of metabolic conservation in your own paper if you’re arguing that this is one of your major contributions.

We appreciate the note here on a potential overstatement of our results. We have clarified now that we have not explicitly tested metabolic consumption, but rather outline a response-specific functionality that could potentially reconciles observed lateralization of EDA outputs with the canonical function of the ANS. See lines 295-299.

Line 170: arm or hand?? Be specific on EDA electrode placement! A figure would be helpful.

Hand. We thank the reviewer for noting this. Stimulator electrodes were placed on the forearm, while recording electrodes were places on the fingers. As part of a new methods figure, we now include a full visualization of our electrophysiological recording set up. See Figure 1.

Line 179-182: “this work demonstrates that heterogeneity of ANS outputs extends beyond differential signaling to separate effector organs, as it also includes differential signaling across body-locations within a single effector organ (i.e., skin).” --> Good Thank you.

Line 194: It is unclear what you mean by “homogenous metrics” for cardiac and respiratory outputs, as they are far from homogenous across individuals (different resting heart rates/respiratory rates across individuals, vastly different responses to stimuli and effort, etc.) and are certainly controlled in a dynamic (what you call “heterogeneous” here) way to enable maximal metabolic conservation. The heart and lungs (and almost every major organ) are also jointly controlled by the parasympathetic and sympathetic branches of the nervous system, making them far from “homogenous” (whatever that means) from an ANS point of view. The authors might note that sweat glands are innervated only by the SNS, making the skin a unique organ to analyze SNS activation.

’Homogenous’ in this context was intended to refer to consistent activation/inactivation across the effector organ - i.e., the heart does not pump harder in one ventricle than another, or one lung instructed to push more air - rather than homogeneity of autonomic input. This section has now been revised to clarify our intended meaning. See lines 329-332.

Lines 198-201: “While the neural architecture for limb-specific vascular responding is well established - localized patterns of dilation are well documented during motor activity and exercise (56-58) - it is unclear whether this localized vascular responding can be used by the ANS for motor preparation as well.” --> This sentence is confusing. What do you mean by “vascular responding can be used by the ANS”? Doesn’t the ANS prompt vascular dilation? I am not an expert on vascular dilation, so there may be an afferent signaling pathway I am unaware of, but the sentence is still confusing and the point they are trying to make is not clear.

We appreciate the note on clarity in this section. The statement above did not intend to question whether the ANS can cause vasodilation (as note by the reviewer, this is very well evidenced), but rather whether localized threat-response can promote localized vasodilation in threatened areas, rather than a general vasodilation to all skeletal muscles. While this type of muscle-specific vasodilation is commonly observed during or following exercise, it is unknown if it occurs in preparation of threat response. We have now clarified this section in hopes of being more transparent with our intended suggestion. See lines 332-340.

Lines 202-203: “It is also unclear whether limb-specific cutaneous activity is observed in response to perception of threat through senses other than others, such as the sight of a spider approaching the hand.” --> A few typos make this hard to read and the point could be clearer. The idea of replicating your study using evocative limb-specific visual stimuli - such as a spider approaching one hand versus another - is novel and important. Such a study would decouple the propagation of the electrical signal from the shock from the resultant SCR while maintaining the fear-conditioning study design (which is likely to produce a strong, reliable SCR). However, you need to make this point much clearer in the sentence.

We agree that this sentence, in its previous construction did not reflect the desired content. We have edited this sentence for clarity, and believe that it now aligns to our intended meaning. See lines 334-340.

Line 223: “e.g.” is in a weird spot. Not sure if its unconventional location is supposed to mean something...

This ordering error was due to an auto-formatting from our reference management program. It has been fixed.

Lines 228-230: “these proposals often rely on the interpretation of shared patterns of activity within the brain, rather than shared peripheral outputs.” The meaning is unclear here.

The intended meaning of this clause was to suggest that prior work linking sensory and cognitive effect states focuses on the central manifestation of these states (i.e., what happens in the brain), rather than a common influence over physiological responding. This has now been revised for clarity. See lines 372-374.

Lines 230-232: “where the affective characteristic shared between general and tactile processes is peripheral in nature, yet only biologically sensible in its tactile manifestation.” Meaning unclear.

Additional context for this statement has been added to increase the clarity of the point. See lines 377-380.

Lines 232-235: “Additional work investigating the neural underpinning of the ANS modulation to both sensation provoked and centrally mediated arousals is still required to determine the extent to which these are overlapping processes within the central nervous system.” --> Yes! Good!

Thank you! We are excited by these questions and hope to target them in some of our future work.

Line 240: “Five of the subjects indicated they are left-handed.” Given that handedness may have dominant effects in EDA lateralization, the effect of participants should be further analyzed (with handedness as a confound or as a separate analysis).

Group-wise analyses separating left and right handed participants are now provided (see Lines 242-254). Importantly, no notable differences were found between these populations after considering changes in statistical power due to sample size. Additionally, Figure 4 (formerly figure 2) now identifies all left-handed participants in single-subject analyses.

Line 246: “remaining 50 subjects”. The stats on these final subjects need to be indicated (how many resultant male/female, ages, handedness, etc.)

This information is now included. See Line 132.

Line 253: “placed on bilaterally on” --> typo Fixed.

Line 264: “placed on the middle and index fingers of each hand” --> distal or medial finger placement? Wet electrodes or dry? Was isotonic gel used? Were the electrodes pre-gelled? Were they taped on? (The pressure and stability of the electrode can greatly affect the signal.) You should also mention the size and composition (Ag-AgCl) of the electrodes and the fact that they were wired. I can get some of this information from googling the specific model (great job including that!), but it still should be briefly stated in the text. A figure or photo of the electrode placement and general setup would be helpful.

Electrodes were placed on over the medial phalange, without conductive gel. Velcro straps fixed to the electrodes were used to secure them in place. This information is now included on Lines 153-156. Additionally, a methods figure (Figure 1a) is now included with the manuscript that includes all electrode placement and equipment setup.

Line 265: “electrocardiogram (ECG)” --> pulse rate monitor is more accurate.

This sentence has been edited for precision.

Line 267: “visual task is run” --> was run

Corrected.

Line 272+: A simple figure/graphic of the procedure would be helpful

A full methods figure is now provided. See Figure 1b.

**Line 278-9: “Shock events were spaced 10,000 ms apart and intermixed with an equivalent number of ’No Shock’ trials.” --> Were the shocks regularly spaced/predictably timed? There was no jittering?? Or every 10 sec they either got a shock or no shock, and they didn’t know which?? Please clarify, as these details are critical for any experiment using a strong orienting stimulus like a shock (or loud noise, etc.).

The latter interpretation here is correct. Every 10 s the participant received either a shock or no shock (randomized), but had no indication of which it would be. The equal number of ’no shock’ events acted as the jitter, and EDA from these trials were measured to create a ’no shock’ condition (See figure 1). This section has now been updated to more clearly reflect the experimental design. See lines 173-177.

Lines 291-294: “manual filter of unlabeled trial-by-trial data was conducted to identify a GSR threshold beyond which the data was most likely attributed to noise in the signal. GSR thresholds were identified independently by two unique raters.” --> Need to describe this process better. What was the “threshold” you used? What was the most common/likely source of noise?

Thresholds were manually determined for each electrode channel (i.e., an independent threshold for both the right and left hand of each participant). Potential sources of noise in the signal include motion-related artefacts, or electrical noise. These points are now clarified from lines 195-202. Additionally, a new figure (Figure 2) has been included that shows data from all trials pre- and post- manual filtering.

**Line 296+: “z-score standardization” --> It is not clear what is being z-scored. Is it the SCR peak? How were the peaks found (e.g., peak fitting function in some program? manually?)? Are the peaks normally distributed (which would be necessary for z scoring)?? Did you normalize the peak height with respect to individual Or how did you deal variation?? with the inherent signal variation across participants (i.e., participants with a range 0-1 microSiemens vs. 0-20 microSiemens)?

Z-scoring was performed on the raw values for each electrode for all samples of each participant prior to any electrode comparison. No peak extraction was performed as all subsequent analyses were conducted on the full time series data (i.e., all inferential statistics were conducted at each sampled time point). These is now clarified from lines 202-205.

Line 296: “by GSR electrode” --> by a pair of GSR/EDA electrodes

Z-score normalization was performed on each GSR electrode independently, prior to calculating the lateralization bias (see above). This is clarified at line 202-209.

**Line 297-300: “For each independent event (i.e., all shock and no shock events), we calculated the right hand GSR - left hand GSR for each time point, resulting in a continuous ’Lateralization Bias’ index for each event” --> This line is unclear. A continuous Lateralization Bias suggests you are using raw values, but then you are referring to some sort of “event”. Is that the stimulus/shock? Or the peak SCR?? Please clarify.

"Each event” in this sentence was intended to refer to each trial, and not each EDA response. This has been corrected for clarity. See lines 205-209. This procedure enabled us to investigate how lateralization biases unfolded over the full time course in each condition (see Figure 3d).

Line 304-5: with all resultant p values subject to a false discovery rate correction.” --> Tell me what the p-value (alpha) was before and after correction.

All p values were corrected to 0.05, and the values reported are those observed following the correction. This is now clarified on line 227-229, prior to presenting the results. The specific uncorrected p-value corresponding to an FDR corrected value of 0.05 depends on the analysis being corrected, For example, in our current Lateralization Bias analyses (a series of one-sample t-test against a comparison value of 0, see Figure 3b) the correspond uncorrected p value is ∼ 0.01.

The following figures would be helpful:

- A figure showing the EDA and stimulus (shock) electrode setup. In particular, please indicate where on the palms/fingers the EDA electrodes were placed, showing the stimulus electrodes in the same photo for scale. A wider photo showing how the participants were seated and/or the general room setup would also be helpful for reproducibility. Include other concurrent measurement equipment, such as the thumb pulse-ox monitor, wires, screens, etc.

A methodology figure is now provided, including participant set-up and experiment conditions. See Figure 1.

- A figure showing examples of raw EDA signals from a few participants. This will help other readers, especially those less familiar with EDA, to get a sense of the scale and variability of an EDA signal. Include at least two representative examples, and noting the variability across individuals, such as “low responders” with signals staying below 2-3 uS versus those whose peaks reach 20 uS.

A new figure (Figure 2) is now available that displays all EDA recording, pre and post manual filtering, for all participants.

Thank you for all of your hard work! I look forward to reading the revised paper!
